# Lightweight 3D Hierarchical Metamaterial Microlattices

**DOI:** 10.1002/advs.202410293

**Published:** 2025-05-02

**Authors:** Luke Mizzi, Krzysztof K. Dudek, Andrea Frassineti, Andrea Spaggiari, Gwenn Ulliac, Muamer Kadic

**Affiliations:** ^1^ Department of Sciences and Methods for Engineering University of Modena and Reggio Emilia Reggio Emilia 42121 Italy; ^2^ Institute of Physics University of Zielona Gora ul. Szafrana 4a Zielona Gora 65‐069 Poland; ^3^ Institut FEMTO‐ST Université de Franche‐Comté CNRS Besançon 25030 France

**Keywords:** auxetic metamaterials, hierarchical systems, lightweight structures, microlattice structures, two‐photon lithography

## Abstract

Hierarchical auxetic metamaterials are a class of materials which are characterized by a multi‐tiered architecture and have the capability of exhibiting enhanced mechanical properties in comparison to their single‐geometry counterparts. In this work, three distinct new classes of hierarchical auxetic metamaterials designed by incorporating cubic crystal lattice geometries, namely, Body‐Centred Cubic (BCC), Face‐Centred Cubic (FCC) and Tetrahedral Cubic (TC) are proposed into 3D rotating cube structures. Through the introduction of hierarchy, these relatively dense mechanical metamaterials are rendered lightweight, through a volume fraction reduction of over 90% in the majority of cases from their full‐block (FB) counterparts, while retaining their original auxetic capabilities. These systems are also demonstrated to possess the ability to exhibit a wide range of stiffnesses and Poisson's ratios, including giant negative values, as well as superior stiffness/density ratios making them ideal for implementation in lightweight applications. Furthermore, a two‐photon lithography 3D‐printer is used to fabricate these new lattice structures at the microscale and test them in‐situ. The results obtained provide clear and comprehensive evidence of the improvement imparted through the introduction of hierarchy and the advantages of using this method to design lightweight 3D rotating unit auxetic structures.

## Graphical abstract

In this work, hierarchical lightweight 3D auxetic metamaterials based on cubic crystal lattice truss geometries are proposed. Microscale prototypes are fabricated using two‐photon lithography additive manufacturing and tested in situ. These hierarchical metamaterials are shown to exhibit a wide range of negative Poisson's ratios including giant auxeticity. The stiffness/density ratio of these metamaterials is superior to non‐hierarchical systems and a volume fraction reduction in the range of 90% can be obtained through the introduction of hierarchy.

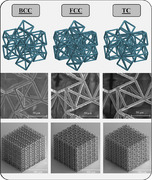

## 1 Introduction

Mechanical metamaterials^[^ ^1^ ^]^ are a class of advanced materials which possess mechanical properties that can be tailored as a function of their structure/geometry. This characteristic imparts a great deal of versatility and customization potential to these systems since one may obtain a desired set of mechanical properties simply by altering the geometry of the metamaterial structure. The geometry‐dependence of these systems also allows for the attainment of anomalous negative mechanical properties such as negative Poisson's ratio,^[^ ^2^, ^3^, ^4^, ^5^, ^6^ ^]^ negative stiffness,^[^ ^7^ ^]^ negative thermal expansion^[^ ^8^ ^]^ and negative compressibility.^[^ ^9^ ^]^ These properties, which are very rarely found in nature, have the potential to exhibit several advantageous characteristics which make them superior with respect to conventional materials, i.e., materials possessing “positive” mechanical properties.

Auxetic metamaterials (negative Poisson's ratio metamaterials) are the most well‐known group of mechanical metamaterials. Since the initial pioneering work by Lakes and Evans over 30 years ago involving the synthesis of auxetic foam^[^ ^10^ ^]^ and design of re‐entrant honeycomb‐based poly‐phenylacetylene networks,^[^ ^2^, ^11^ ^]^ there have been several advancements in the field leading to the discovery of a variety of deformation modes and geometries which give rise to auxeticity as well as the implementation of these systems in a wide range of applications. These uses, which typically involve niche applications requiring specialized properties characteristic of auxetic metamaterials such as ability to undergo synclastic curvature,^[^ ^12^, ^13^ ^]^ high indentation resistance^[^ ^14^ ^]^ and large deformation capabilities,^[^ ^15^, ^16^, ^17^, ^18^ ^]^ as well as enhanced energy absorption potential,^[^ ^19^ ^]^ include personal protection equipment,^[^ ^20^ ^]^ flexible electronic devices^[^ ^21^ ^]^ and biomedical implants^[^ ^22^ ^]^ amongst others (Supplementary Figure S1, Supporting Information).

Auxetic metamaterials have also been identified as promising candidates for implementation in devices and structures requiring lightweight, high‐porosity systems with an appreciable load‐bearing capacity. Rotating unit mode metamaterials,^[^ ^23^, ^24^ ^]^ are of particular interest in this regard. As the name implies, this group of metamaterials is characterized by a deformation mode upon loading which involves the rotation of rigid or *quasi*‐rigid blocks of material. It includes several configurations which have the potential to exhibit auxetic behavior such as rotating squares,^[^ ^23^, ^25^ ^]^ triangles,^[^ ^24^ ^]^ rectangles,^[^ ^26^ ^]^ parallelograms,^[^ ^27^, ^28^ ^]^ and cuboids,^[^ ^29^, ^30^, ^31^ ^]^ as well as multiple polygons,^[^ ^32^, ^33^, ^34^, ^35^, ^36^, ^37^ ^]^ and hierarchical arrangements.^[^ ^15^, ^38^, ^39^, ^40^, ^41^ ^]^ This class of auxetic metamaterials is also known to exhibit the highest stiffness (effective Young's modulus) in comparison to other metamaterial groups by a considerable margin. This relatively high stiffness, however, is offset by a significantly high material volume fraction (i.e., high density; low pore volume ratio) as well as a low strain tolerance and fatigue performance.^[^ ^42^ ^]^ Obtaining the ideal balance between these contrasting characteristics can be a challenge, yet, an achievable one since they are all governed by geometry and thus, through smart design, one may attain a set of properties which satisfy the desired requirements for a specific application (Supplementary Video S1, Supporting Information).

**Figure 1:**
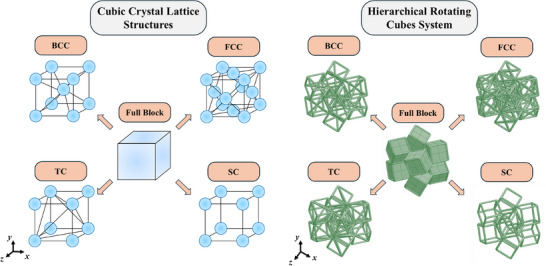
Schematic showing cubic crystal lattice arrangements and their implementation for the design of hierarchical rotating cube metamaterials. The structures on the right show the representative volume elements of the full block and four hierarchical structures proposed in this work.

The introduction of structural hierarchy is one route through which this may be achieved. Hierarchical metamaterials are systems which are characterized by a multi‐level geometry which imparts superior mechanical characteristics to the structure with respect to a single level metamaterial.^[^ ^43^, ^44^, ^45^, ^46^, ^47^, ^48^ ^]^ The addition of hierarchical elements increases the versatility of the system and also allows for a more efficient design of the metamaterial with respect to the desired mechanical properties and other accompanying characteristics, such as improved stiffness/density ratio^[^ ^45^ ^]^ or higher strain tolerance.^[^ ^49^, ^50^ ^]^ In this work we have applied this concept to obtain a new class of 3D lightweight hierarchical auxetic metamaterials. These systems are primarily based on single level rotating cube structures which are then evolved into truss‐based hierarchical systems inspired by cubic crystal lattices.^[^ ^51^ ^]^ Various hierarchical forms such as Body‐Centred Cubic (BCC), Face‐Centred Cubic (FCC) and a custom tetrahedral cubic arrangement (TC) were employed to reduce the density of metamaterial structure (see **Figure** **1**) whilst retaining the negative Poisson's ratio and an acceptable level of structural stiffness. The rationale behind this approach was that the utilization of stiff lattice‐based truss configurations to form the rotating cube units would impart superior stiffness/density ratios to these metamaterials along with lower volume fractions and improved porosity while retaining the original rotating mechanism from which auxeticity arises in these metamaterial systems. In addition, a bending‐dominated Simple Cubic (SC) form was also designed and evaluated in order to evaluate the influence of the introduction of a relatively weak hierarchical structural framework and allow for comparison with the aforementioned stiffer stretch‐dominated architectures. These hierarchical metamaterial structures were evaluated using an extensive parametric approach utilizing numerical methods followed by experimental tests on microstructural prototypes produced by two‐photon lithography. The results obtained demonstrate that this design method is an extremely promising and viable approach which may be used to obtain 3D lightweight hierarchical auxetic metamaterials.

## 2 Concept and Design

The rotating cubes system is made up of solid cubes, each of which is connected to six adjacent ones: four at the vertices and two at the edges.^[^ ^29^ ^]^ The representative volume element (RVE) of the metamaterial system is made up of eight cubes as shown in Figure 1a and possesses three planes of mirror symmetry. In its idealized form, the cubes are connected together through spherical joints allowing for rigid rotation of the units relative to one another; however, in single‐material rotating unit systems the cubes overlap each other at the vertices and edges resulting in an interconnection region which absorbs the bulk of deformation upon loading and allows the system to deform in a manner which corresponds roughly to the idealized variant.

This system is predicted through analytical models to exhibit a negative Poisson's ratio in each of the three principal on‐axis planes and its Young's modulus is primarily determined by the stiffness of the idealized hinges or volume and shape of the material at the interconnection regions. This is due to the fact the cube unit is assumed to be undeformable and can only undergo rigid body rotation. However, this is not strictly necessary in order for the system to exhibit auxetic behavior. Provided that the overall shape of the cube is retained upon loading (i.e., uniform contraction or expansion) the metamaterial system can still exhibit a negative Poisson's ratio through a mixture of unit rotation and localized deformation. This means that, provided that the correct internal hierarchical geometric arrangement is chosen for the cubes, one should be able to significantly alter the stiffness and density of the system whilst leaving the Poisson's ratio unchanged. This concept has already been demonstrated to be functional in 2D truss‐based hierarchical rotating unit structures,^[^ ^45^ ^]^ however extending this concept to 3D systems is expected to provide a number of additional challenges in view of the increased complexity of the geometry and added number of degrees of freedom imparted by the introduction of hierarchy to a 3D system.

**Figure 2:**
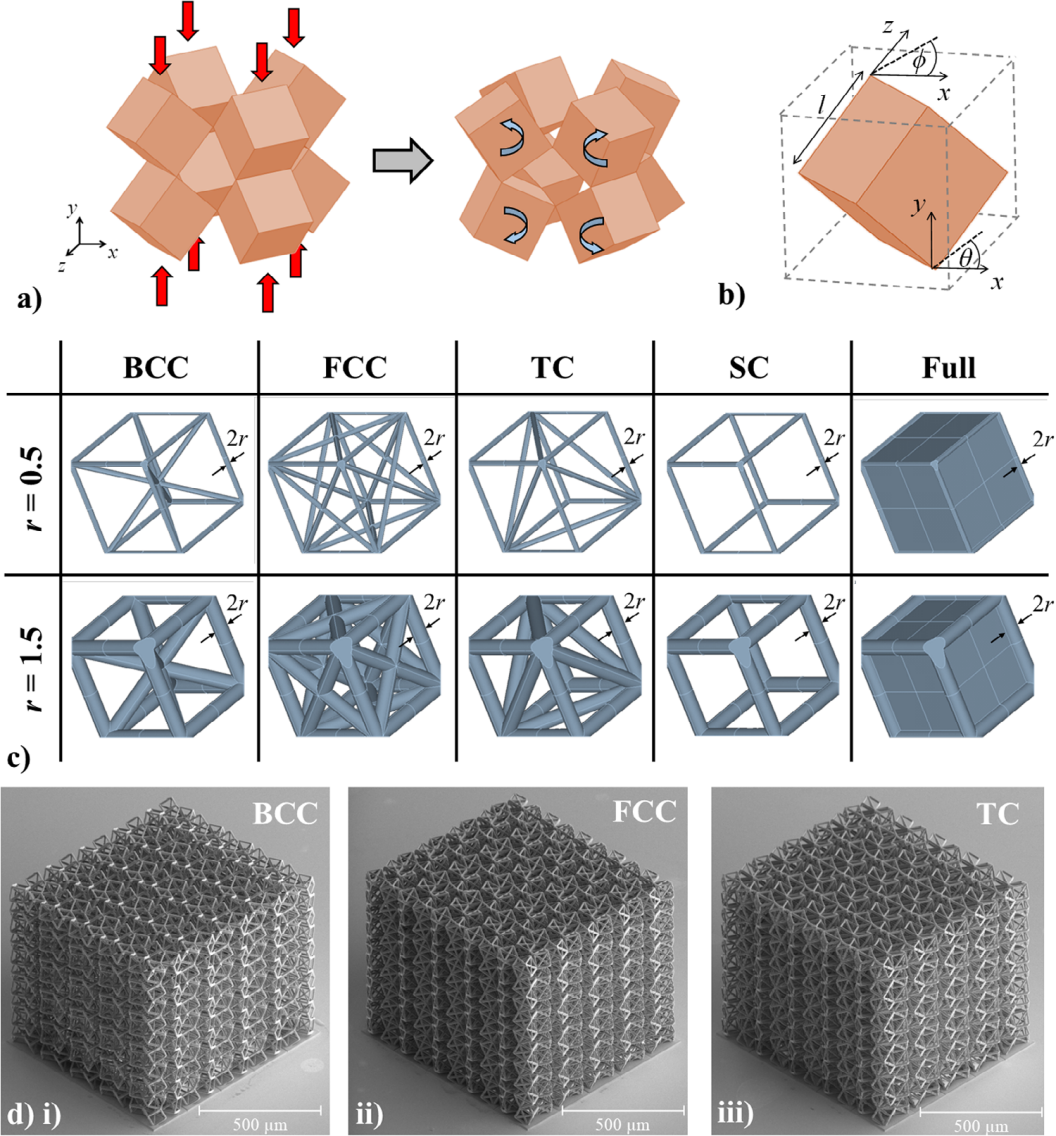
a) An idealized rotating cubes representative unit cell deforming under compressive loading and b) 1/8^th^ of the unit cell showing the geometric parameters *l*, *θ* and *ϕ* used to define the system. c) Images showing the realistic simulated systems with the ligament/interconnection thickness parameter, *r*, for the five configurations investigated. d) SEM images of the 5×5×5 BCC, FCC and TC lattice structures fabricated using two‐photon additive manufacturing.

In order to obtain lightweight auxetic metamaterials, we incorporate a hierarchical truss system based on cubic crystals. The four forms chosen are presented in Figure 1b and correspond to SC, BCC, FCC and TC. In order to analyze the mechanical properties of these structures, we conducted a wide ranging Finite Element Method (FEM) parametric study on a large variety of systems. As shown in **Figure** **2**b, the idealized generic rotating cube system may be defined in terms of three variables: the cube length dimension, *l*, and the angular rotations in the *xy* and *xz* planes denoted as *θ* and *ϕ* respectively. In practical terms, another geometric variable is necessary to define the interconnection region between cubes. In this case, the truss networks were based on circular tubes with a spherical end‐closer as shown in Figure 2c and thus the interconnection parameter may be described in terms of the radius of this sphere, *r*. This parameter may, in turn, also be used to describe the thickness of the cylindrical cross‐section trusses used to realize the hierarchical structures as shown in Figure 2c. In this work, the parameter *l* was kept constant at a value of 30 µm, while the parameters *θ* and *ϕ* were both altered separately from a range of 5–85° in steps of 5°. This is in line with the theoretical limits for both these angles, which are between 0° and 90°. In addition, the ligament and interconnection radius, *r*, was changed from 0.5 to 2 µm. These four parameters were used to produce the four truss configurations shown in Figure 2c as well as the full, rotating cube equivalent of each of these structures (which was also analyzed for sake of completeness and to allow for a comparative analysis). In the latter case, the parameter *r* defines only the radius of the interconnection sphere since no ligaments are present. The entire parametric run covered in this study comprises a cumulative number of 5915 simulated systems; a relatively high number which is essential in order to obtain an all‐encompassing view of the potential of this new class of hierarchical metamaterials.

To minimize computational time, we took advantage of the planes of axial mirror symmetry which define the eight‐cube representative volume element (RVE) of these metamaterials and simulated the systems as a single rotating unit with mirror boundary conditions.^[^ ^52^ ^]^ Further details on the simulation methodology used is provided in the Experimental Section at the end of the article. Following the initial parametric simulation, a select number of these new hierarchical metamaterials were realized at the microlevel in the form of 5×5×5 lattice structures (see Figure 2d) using a Nanoscribe two‐photon lithography 3D printer and tested in situ using uniaxial *quasi*‐static compressive loading tests coupled with Digital Image Correlation (DIC) analysis to investigate their mechanical properties. Further information on the fabrication of these systems, which were produced with a ligament thickness of 6 µm (i.e., *r* = 3 µm) and an overall gauge length less than 1 mm, is also provided in the Experimental Section.

## 3 Results and Discussion

**Figure 3:**
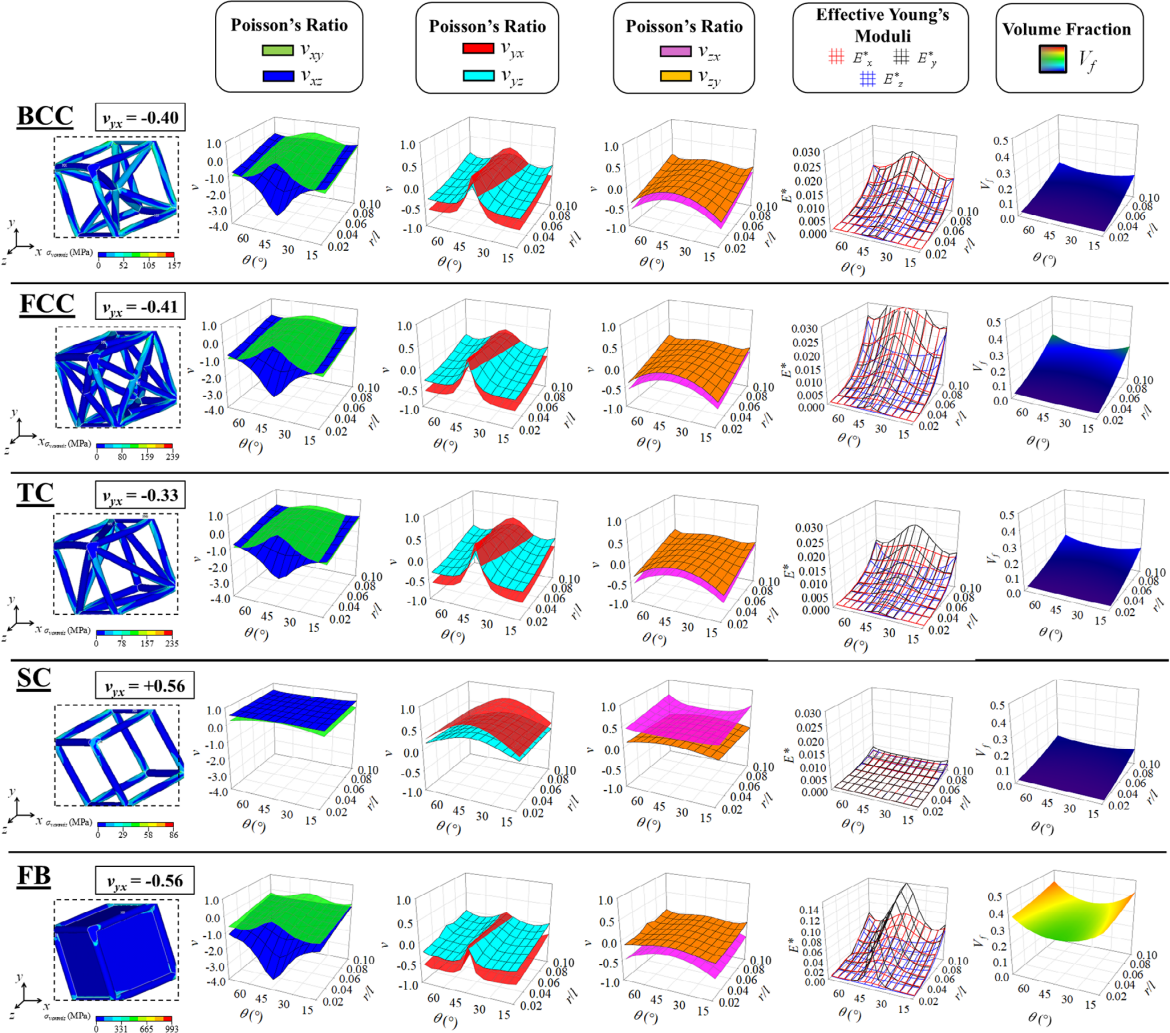
Images showing the deformed structures at 5% compressive strain (using magnified displacement scaling) applied in the *y*‐direction for the BCC, FCC, TC, SC and FB systems with *r*/*l* = 0.05, *ϕ* = 30° and *θ* = 30°. Plots are also provided showing how the mechanical properties and volume fractions of these systems vary with changing *r*/*l* ratio and *θ* values. The parameter *ϕ* was kept constant at 30° for all the plots shown here; the full data set is provided in the Supporting Information.

A representative sample of the results obtained for the five sets of metamaterial structures is presented in **Figure** **3**. It is clearly evident even from the relatively small set of results presented (only systems with *ϕ* = 30° are shown, representing 1/13 of the covered factorial space – full results can be found in Supporting Information), that these systems have the potential to exhibit a wide range of mechanical properties. The systems are highly anisotropic and, in the cases presented in Figure 3, the Poisson's ratios vary from +0.5 to ‐4.0, with the most negative values being observed in the *xz*‐plane. There is also significant variation in the effective Young's moduli, *E* ^*^, with the highest stiffness being consistently obtained for loading in the *y*‐direction. This is a unitless parameter, which defines the stiffness of the system in terms of the geometry independently of the material used, and is calculated as follows: *E* ^*^ = *E*/*E_mat_* where *E* is the metamaterial absolute Young modulus and *E_mat_* is the material Young modulus. The volume fraction of these systems, which is calculated as the metamaterial real volume divided by the RVE volume, is also plotted.

It is clearly evident that the trends obtained for the Poisson's ratios and Young's moduli of the hierarchical FCC, BCC and TC systems are extremely similar to those of non‐hierarchical FB systems despite the significant reduction in volume fraction. This demonstrates that in spite of the introduction of these hierarchical configurations, the overall deformation mechanism which imparts auxeticity, i.e., the rotating mechanism, remains active. On the other hand, in the case of the SC systems, completely different trends were observed leading to a loss of auxeticity and an overwhelming plummet of the global stiffness. This indicates that the SC arrangement results in structural deficiencies which makes other deformations such as the collapse of the cubic unit itself more favorable with respect to *quasi*‐rigid rotation, hence the complete change in trends observed for the mechanical properties. This clearly shows that this hierarchical arrangement is not suitable for the design of auxetic lattice structures of this type.

In terms of Poisson's ratios, the most negative values were generally observed for the FB systems. However, the FCC and BCC systems both exhibit extremely similar values, while the TC systems showed Poisson's ratios with a slightly reduced magnitude. On the other hand, for the SC systems every configuration demonstrated a positive Poisson's ratio. This is due to the fact that, as shown in Figure 3, in the SC system, the hierarchical arrangement used to construct the “rotating unit” is not sufficiently robust, thus resulting in a non‐uniform deformation of the ligaments which in turn leads to a “flattening” of the unit rather than semi‐rigid rotation. The concept of semi‐rigid rotation is what the drives the auxetic behavior of the non‐hierarchical FB system and the absence of this deformation mechanism is manifestly evident for the SC geometry. On the other hand, in the FCC, BCC and TC systems a mixture of ligament flexure and semi‐rigid rotating unit is occurring as shown in the von‐Mises stress distribution images in Figure 3. This indicates that even in these cases the rotating unit does not remain rigid. However, while for the SC systems, flexural deformation of the ligaments making up the hierarchical rotating unit results in a complete loss of auxeticity, for the FCC, BCC and TC metamaterials only a marginal increase in Poisson's ratio is observed. This is due to the fact that FCC and BCC lattice structures are classified as stretch‐dominated periodic systems according to the Maxwell Criterion^[^ ^53^ ^]^ and therefore this hierarchical arrangement imparts a high level of structural rigidity to the skeletal rotating cube. The TC system also exhibits a relatively high degree structural stiffness for similar reasons, albeit at a lower value in comparison to the FCC and BCC systems, in view of the presence of longer ligaments for corresponding structures of the same overall global dimensions. The fact that these three configurations are endowed with such a structurally robust hierarchical truss system, means that all ligaments undergo *quasi*‐uniform flexural deformation resulting in the rotating unit retaining its original shape and contracting as a whole upon the application of compressive loading. This means that the negative Poisson's ratio of the system is retained since uniform contraction coupled with rotation of the cubic units results in an overall shrinkage of the system in all directions.

The increased robustness of the FCC, BCC and TC hierarchical geometries in comparison to the SC is also amply demonstrated in the plots showing the variation of effective Young's moduli for these metamaterial systems in Figure 3. All three systems exhibit a far higher relative stiffness with respect to the SC systems, although their effective Young's moduli are still much lower than that of the full, non‐hierarchical corresponding structures. In the latter case, however, this high stiffness, which arise primarily due to the fact that all of the localized deformations are concentrated at the interconnection regions, is offset by an extremely large volume fraction and increased von Mises stress levels.

**Figure 4:**
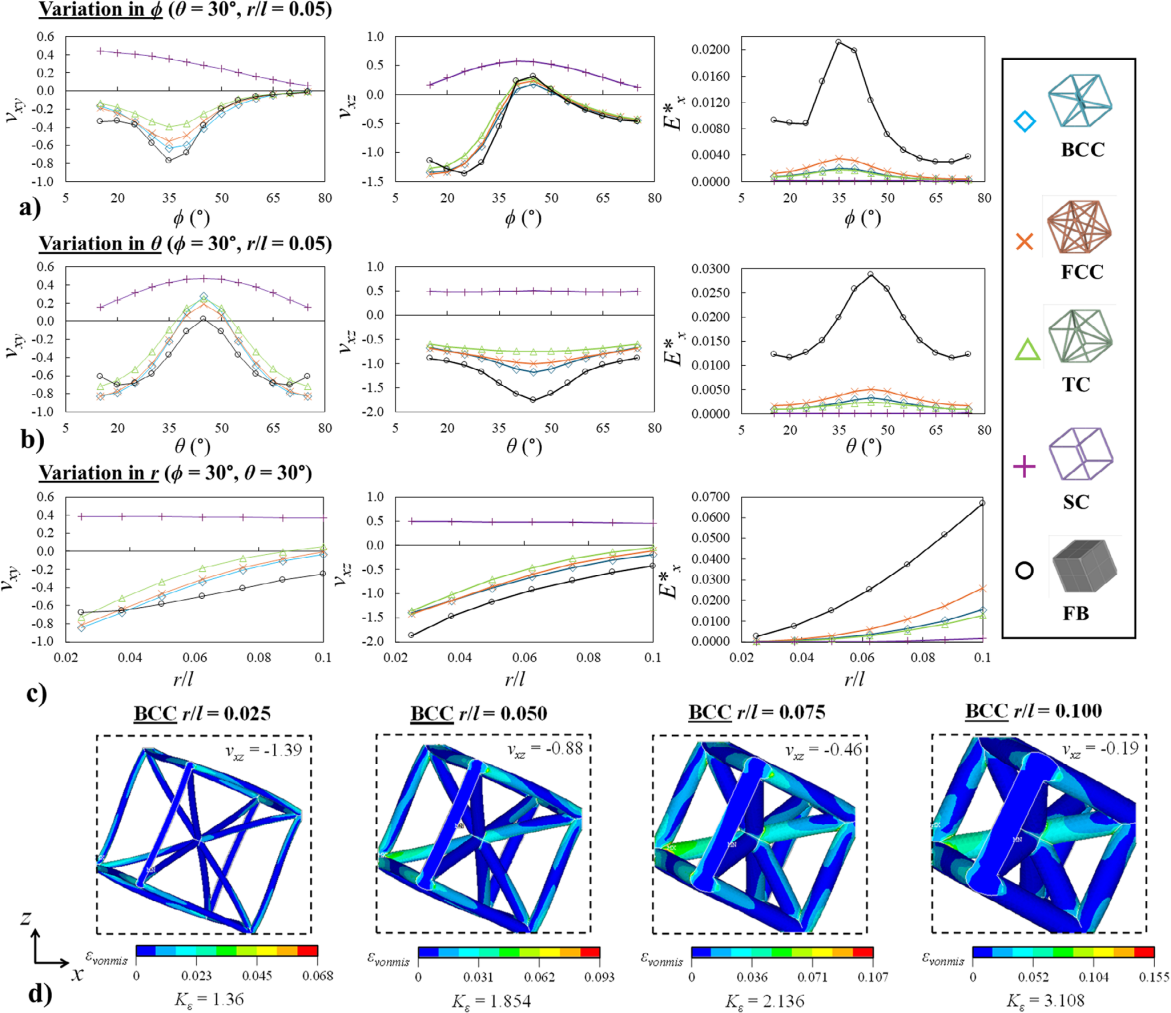
Plots showing how the variation in a) *ϕ*, b) *θ* and c) *r* influences the mechanical properties for loading in the *x*‐direction. d) Images showing the deformed shapes at 5% compressive strain (using magnified displacement scaling) in the *x*‐direction for some of the BCC structures plotted in c) with different ligament radii. The dashed lines indicate the size of the undeformed system. The distribution of localized von Mises strain, *ε_vonmis_*, is shown as well as the Poisson's ratio obtained and strain concentration factor, *K_ε_*.

In order to allow for a more extensive comparative analysis of these systems, a representative set of structures of each class is presented in **Figure** **4**, showing how the mechanical properties for loading in the *x*‐direction, i.e., Poisson's ratio in the *xz*‐plane and *xy*‐plane, *ν_xz_* and *ν_xy_*, and the Young's modulus in the *x*‐direction, *E_y_*, vary by changing solely as single geometric parameter. It is manifestly clear from these plots that, as stated previously, the hierarchical BCC, FCC and TC systems exhibit extremely similar Poisson's ratios to the full block non‐hierarchical equivalents while the SC systems exhibit completely different mechanical properties. This entails a retention of the original versatility of the rotating cubes mechanism, since, as shown in Figure 4a,b, the large variations in Poisson's ratio and overall trends as a result of changing the angles *ϕ* and *θ* are preserved, albeit at a slightly lower magnitude for the most part. However, in some case where one of these two angles has a low value, i.e., 15°, the Poisson's ratio of the hierarchical systems is actually lower than that of the non‐hierarchical equivalent. This is due to the fact that in these cases, where the system is already close to its fully‐closed configuration and is being subjected to compressive loading, the presence of deformable ligaments instead of a rigid block of material in the rotating unit provides the metamaterial system an alternative deformation mode (which also leads to auxetic behavior) besides semi‐rigid rotation. In terms of effective Young's moduli, it is evident that the FB systems exhibit a considerably larger stiffness in comparison to the corresponding hierarchical stiffness, typically in the range of one order of magnitude higher. This was to be expected, since it is well‐known in the literature that non‐hierarchical systems are always significantly stiffer than their hierarchical counterparts (i.e., one cannot reinforce a structure through the introduction of holes/pores). From the range of hierarchical structures evaluated, the FCC configuration was demonstrated to consistently possess the highest effective Young's modulus, followed by the BCC, TC and SC systems respectively, with the latter exhibiting a considerable drop in stiffness in comparison to the other three configurations.

Another important parameter to consider is the radial thickness dimension, *r*, which can also be referred to as the *r*/*l* ratio since *l* was kept constant at all times and thus a change in this ratio represents only a change in *r*. As shown in Figure 4c and r/*l* has a profound influence on the mechanical properties of all systems except for the SC structures. BCC, FCC, TC and FB systems with a low *r* value possess the most negative Poisson's ratios, which becomes increasingly less auxetic as the value of this parameter increases. In terms of deformation mechanism, the influence of this parameter is also evident in Figure 4d, where the results obtained for the BCC system are shown. While for the structure with a small *r*/*l* value, the deformation mechanism is characterized by marked flexure of the ligaments and rotation of the hierarchical unit, these deformation modes gradually disappear as the ratio increases resulting in a less negative overall Poisson's ratio. This is due to the fact that thick ligaments are considerably less amenable to flexural deformation than thin ones, while the additional thickening of the interconnection region also inhibits the rotational mechanism. These inferences are also confirmed by the trends observed for the effective Young's moduli. A marked increase in stiffness is observed upon increasing the value of *r*, which rises in a non‐linear exponential manner. This increase in effective Young's modulus is also accompanied by an increase in the strain concentration factor, *K_ε_*, highlighting the difficulty of the system in deforming via the same manner as in its corresponding thin ligament and small interconnection region counterpart. The latter is a dimensionless parameter which is obtained by dividing the maximum localized von Mises elastic strain with the globally applied strain (0.05 in this case). The higher this value is, the more likely the metamaterial structure is to undergo failure at low strains and, thus, by comparing the relative values of this constant, it is possible to identify which configurations have greater strain tolerance with respect to one another through a comparative analysis. In the cases of the systems presented here, it is evident that while the effective Young's modulus increases upon increasing *r*, at high values, this comes at a cost of a loss of auxeticity and a significantly decreased strain tolerance (i.e., a higher *K_ε_* value).

**Figure 5:**
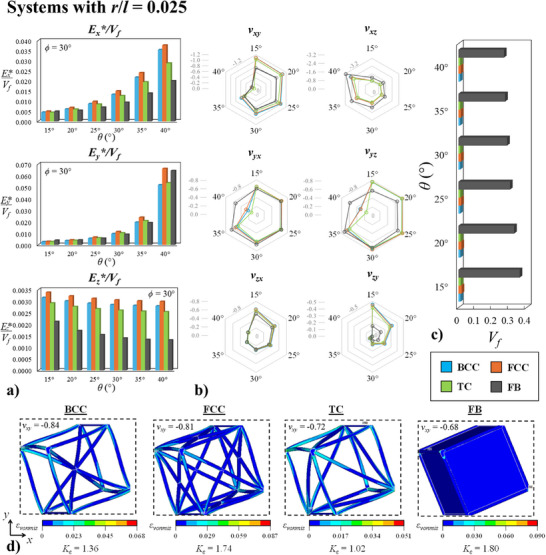
Data and results obtained for a representative set of systems with *r*/*l* = 0.025. a) Plots showing how the variation in the E*/*V_f_* ratio for loading in the *x*, *y* and *z*‐directions for systems with *ϕ* = 30° and varying *θ* values. The data ranges from *θ* = 15° to 40° only since as shown in Figure 4b, the systems *θ =* 50° to 75° show exactly the same mirroring values and the *θ =* 45° value represents a singularity in the deformation mechanism. b) Plots showing the Poisson's ratios of these structures and c) a graph showing the volume fraction of each structure. d) Images showing the deformed shapes at 5% compressive strain (using magnified displacement scaling) in the *x*‐direction for the *θ* = 30°, *ϕ* = 30° BCC, FCC, TC and FB systems. The dashed lines indicate the size of the undeformed system. The distribution of localized von Mises strain, *ε_vonmis_*, is shown as well as the Poisson's ratio obtained and strain concentration factor, *K_ε_*.

This point brings us to one of the main motivations behind the design of these hierarchical metamaterial structures; the possibility of obtaining metamaterial systems which retain their auxeticity whilst possessing enhanced stiffness/density ratios in comparison with their corresponding non‐hierarchical counterparts. Keeping this ratio at a low value has traditionally always presented a number of design problems in the case of rotating unit mode auxetic metamaterials since the “rigidity” of the material block is obtained through its densification, thus resulting in an increased mass and density of these metamaterials in comparison to other truss‐deformation‐based auxetic structures such as re‐entrant and chiral honeycombs. As shown in **Figure** **5**, the BCC, FCC and TC hierarchical structures present a significant step toward solving this problem. Given that the SC system has been demonstrated to suffer from a catastrophic loss of stiffness as well as auxeticity, this geometry will not be considered further in the subsequent analysis.

In Figure 5a, the effective Young's moduli to volume fraction ratio, E*/*V_f_*, for a representative set of systems with fixed *ϕ* = 30° and *r*/*l* = 0.025 with variable *θ* values are presented. This ratio is a modified corresponding version of the traditional stiffness/density coefficient which eliminates the material aspect from the metamaterial structure and considers it as a standalone geometry.^[^ ^42^ ^]^ It is clearly evident that in almost all cases the hierarchical metamaterials exhibit superior ratios in comparison to the full block systems, with the FCC systems showing the best performance followed by the BCC and TC structures, respectively. This enhanced ratio is also obtained whilst retaining a comparable Poisson's ratio to full block system, as shown in Figure 5b, and includes even giant negative values lower than −2. In some cases, such as *ν_xy_*, *ν_zx_* and *ν_zy_*, the hierarchical systems are shown to consistently possess even more negative values than their corresponding FB systems. The main exception to this trend appears to be the system with *θ* = 40° for loading in the *y*‐direction, although this was to be expected since in this particular case the structure is extremely close to its fully‐opened configuration in the *y*‐direction and is, hence, characterized by a relatively extremely high E*_y_ value due to the fact the rotating mechanism is partially block when loading in this direction.

It is also worth mentioning that there is a vast discrepancy in the actual volume fraction, *V_f_*, of hierarchical systems versus the FB structures. As shown in Figure 5c, the FB systems possess a *V_f_* which is roughly one order of magnitude higher than that of the corresponding FCC, BCC and TC systems, which means that, provided that all systems are made from the same constituent material, the hierarchical geometries have 1/10th the density of the FB systems making them particularly well‐suited for lightweight applications. It is also evident from Figure 5d, that the FCC, BCC and TC metamaterials show reduced localized strain concentration factors due to the distribution of strains along the ligaments, indicating that these systems should possess an increased global strain tolerance in comparison to the non‐hierarchical FB structures.

**Figure 6:**
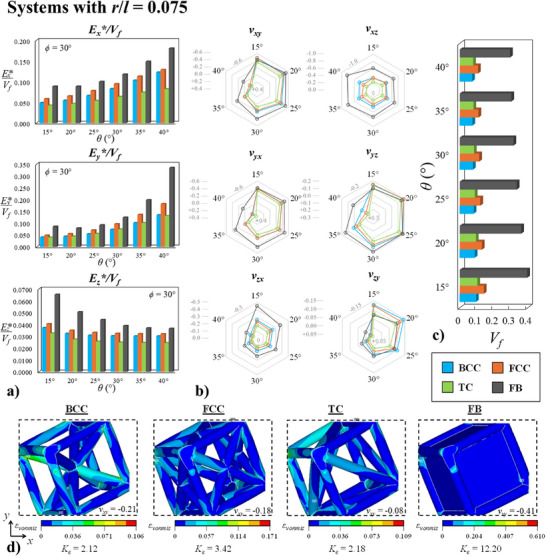
Data and results obtained for a representative set of systems with *r*/*l* = 0.075. a) Plots showing how the variation in the E*/*V_f_* ratio for loading in the *x*, *y* and *z*‐directions for systems with *ϕ* = 30° and varying *θ* values. b) Plots showing the Poisson's ratios of these structures and c) a graph showing the volume fraction of each structure. d) Images showing the deformed shapes at 5% compressive strain (using magnified displacement scaling) in the *x*‐direction for the *θ* = 30°, *ϕ* = 30° BCC, FCC, TC and FB systems. The dashed lines indicate the size of the undeformed system. The distribution of localized von Mises strain, *ε_vonmis_*, is shown as well as the Poisson's ratio obtained and strain concentration factor, *K_ε_*.

However, the efficiency of the hierarchical designs decreases significantly upon increasing the *r*/*l* ratio. As shown in **Figure** **6**, where corresponding plots relating to systems with *r*/*l* = 0.075 are illustrated, the trends shift completely. Now, in each case, the FB systems perform better, exhibiting significantly higher E*/*V_f_* ratios for loading in every in‐plane direction as well as more negative Poisson's ratios in comparison to their BCC, FCC and TC counterparts. The difference in volume fraction between the hierarchical and non‐hierarchical systems is also considerably lower, with the latter metamaterials showing on average 25% of the *V_f_* of the FB systems (see Figure 6c). At this point, however, it is worth noting that the rotating mechanism from which the auxeticity of these systems is derived, is extremely restricted at this *r*/*l* ratio. In fact, the overall range of negative Poisson's ratios, observed in Figure 6b, is nowhere near that shown by the thin ligament/interconnection systems shown in Figure 5. The most auxetic structure exhibits a Poisson's ratio, *ν_xz_*, of −0.8, which is extremely unremarkable when compared with its corresponding thin ligament/interconnection counterpart of −3.2. Moreover, many configurations show a positive Poisson's ratios for loading in some directions. All of this, coupled with the fact that all of these systems exhibit extremely high *K_ε_* values (see Figure 6d), indicates that due to the thick interconnection region, these metamaterials are unable to deform primarily via semi‐rigid rotation of cubes, and thus the utilization of hierarchical building blocks, which further structurally weaken the rotating cube, merely makes this deformation mechanism even less favorable resulting in a significant loss of both auxeticity and E*/*V_f_* ratios. All of this demonstrates that the hierarchical arrangements employed in this work are highly effective primarily for systems in which the rotating mechanism is the predominant deformation mode, i.e., systems with thin interconnections between cubes.

**Figure 7:**
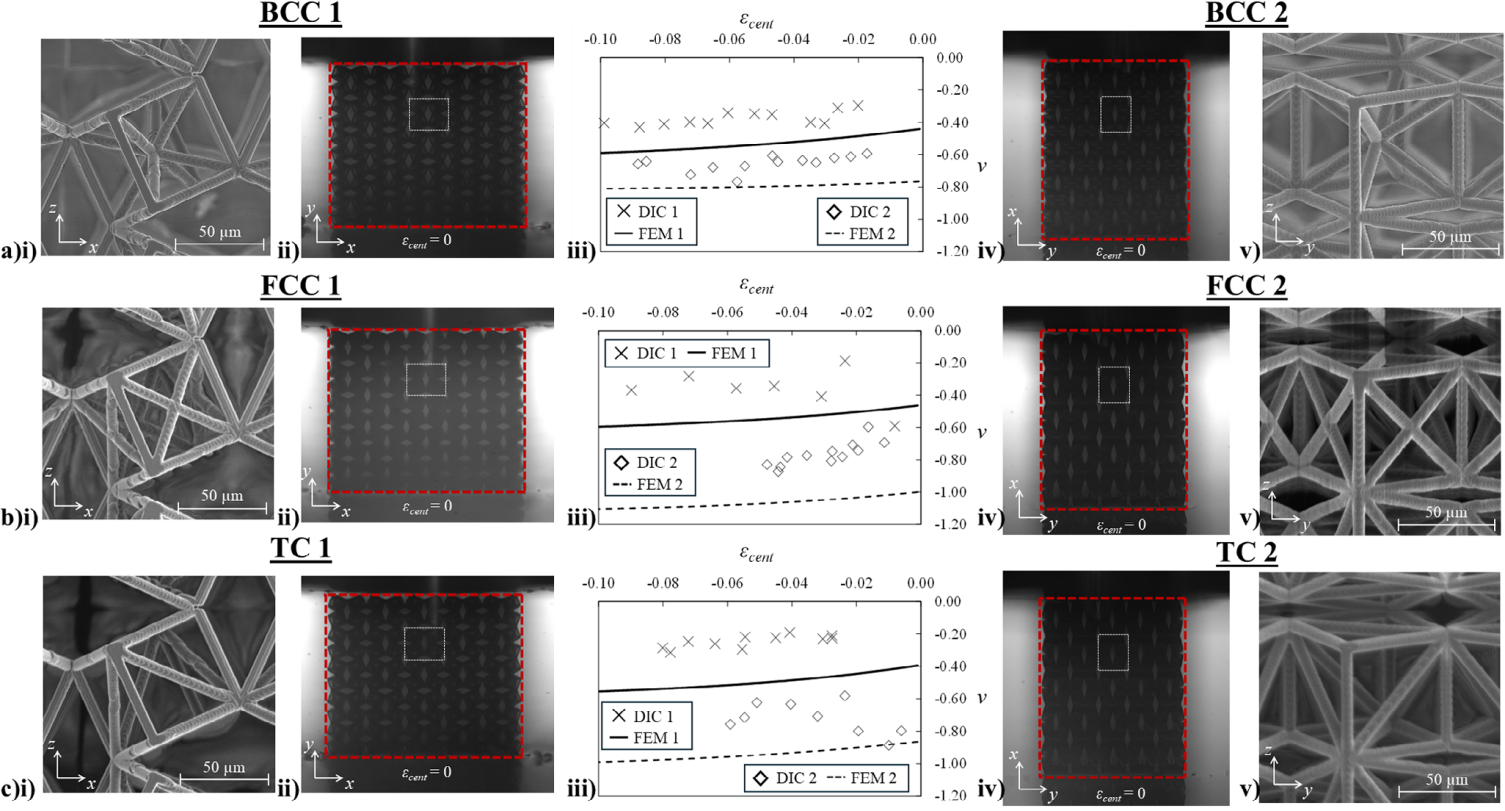
Results of the experimental tests for the a) BCC, b) FCC and c) TC microlattice structures. i) Zoomed‐in SEM image of a single cube for Set 1 systems and ii) optical camera images showing the undeformed global Set 1 systems. The red lines indicate the size of the undeformed lattice structure while the white box indicates the central unit cell from which the plotted results were extracted using DIC. iii) Plots showing how the engineering Poisson's ratio *ν* varies with strain for the central unit cell for the experimental results (DIC) and the corresponding nonlinear numerical simulations on periodic structures (FEM). iv) Optical camera images showing the undeformed global Set 2 systems and v) zoomed‐in SEM image of a single cube for Set 2 systems. Videos of the deformation of the global systems are presented in the Supporting Information.

In order to validate the results and trends obtained from the FEM simulations, six representative microlattice structures pertaining to the BCC, FCC and TC hierarchical class were fabricated in tough resin using a Nanoscribe two‐photon lithography 3D‐printer and tested. These systems were manufactured as 5×5×5 RVE lattice systems (see Figure 1d) and comprised two sets. The first set, Set 1, included BCC, FCC and TC systems with the following parameters: *θ* = 25°, *ϕ* = 25°, *l* = 60 µm and *r* = 3 µm, while the second set, Set 2, contained systems with the dimensions: *θ* = 30°, *ϕ* = 15°, *l* = 60 µm and *r* = 3 µm. These configurations were chosen on the basis of the wide‐ranging parametric simulation run and the design specifications of the additive manufacturing method. Following printing, the systems were subjected to in situ uniaxial compressive loading tests using a flat indenter as shown in **Figure** **7**. Set 1 systems were fabricated in an orientation specifically designed for loading in the *y*‐direction and analysis of the Poisson's ratio *ν_yx_*, while Set 2 systems were oriented for loading in the *x*‐direction and measurement of the Poisson's ratio *ν_xz_*. During testing a high‐resolution optical camera was used to record images which were then used for Digital Image Correlation (DIC) analysis of the deformation of the central unit cell. Note that Poisson's ratio was extracted from the central unit cell in the second row of RVEs due to the fact that the bottom layer of the 5×5×5 lattice is glued to a resinous platform and thus for these types of tests this unit cell is usually considered to be the least affected by boundary effects.^[^ ^47^, ^54^, ^55^ ^]^ In addition to the experimental tests, corresponding systems were also simulated using nonlinear geometric loading conditions, the results of which are presented in Figure 7(iii). Further information on the additive manufacturing process, DIC analysis and nonlinear simulations are provided in the Experimental Section and Supporting Information.

It is clear from the images and plots shown in Figure 7, that these hierarchical systems exhibit a negative Poisson's ratio which is maintained over a relatively high strain range (in some cases up to −10% strain). The magnitude of the Poisson's ratio obtained from the DIC analysis of the experimental results is consistently slightly lower than that of the corresponding nonlinear simulations, however this was to be expected for several reasons. The first is that the latter results consider completely periodic systems, not finite structures, and in the case of the experimental systems, although we consider a central RVE for the DIC analysis, the frontal plane is at the boundary and therefore the RVE under consideration is exposed to significant edge effects. In addition, the SEM analysis of the fabricated systems revealed the presence of structural imperfections such as variations in ligament/interconnection region thickness in the experimental prototypes which probably give rise to significant deviation from the FEM results. However, despite these setbacks, the trends obtained from the experimental results mirror completely those extracted from the numerical analysis. In the case of the Set 1 structures, the BCC and FCC systems exhibit almost identical Poisson's ratios which are both more negative than the corresponding TC system. In the Set 2 systems, which are predicted by FEM simulations to exhibit a more negative Poisson's ratio with respect to Set 1 structures, the FCC system exhibits the most negative Poisson's ratio followed by the TC and BCC systems respectively. Finally, all six hierarchical metamaterials generally exhibit a varying Poisson's ratio which becomes slightly more negative than the initial value as the compressive strain increases. These results confirm these hierarchical systems have the capability of exhibit auxetic behavior similarly to their full block non‐hierarchical corresponding metamaterial structures and that the deformation modes and mechanical properties predicted by the FEM simulations can be replicated at the microscale level. The force‐displacement plots for these structures are also presented in the Supporting Information. It is worth noting that although the central unit cell did not undergo visible fracture, cracking was visible in the lateral columns of RVEs, which, as expected, experience higher localized deformations during loading. This localized failure was evident in the force‐displacement plots as well. This probably also contributed to the lower magnitude of negative Poisson's ratio observed in the experimental results in comparison to the periodic FEM simulations.

**Figure 8:**
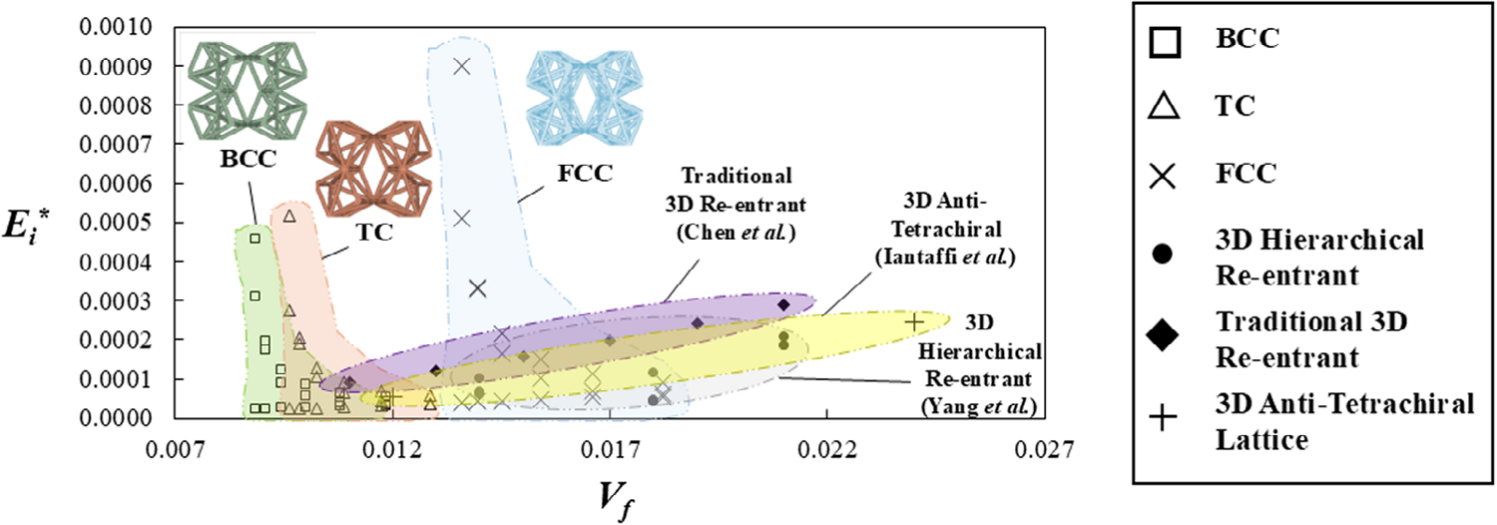
Plots showing how the effective Young's moduli, E_i_^*^, which represents the E_x_^*^, E_y_^*^ and E_z_^*^ values, varies with volume fraction, *V_f_*, for the BCC, FCC, TC and three class of 3D auxetic metamaterials found in literature: a) hierarchical 3D re‐entrant honeycombs proposed by Yang et al.,^[^ ^58^ ^]^ b) 3D anti‐tetrachiral lattices designed by Iantaffi et al.^[^ ^57^ ^]^ and c) the traditional 3D re‐entrant honeycomb investigated by Chen et al.^[^ ^56^ ^]^ The hierarchical rotating unit set comprised systems with *r*/*l* ratios of 0.025, *ϕ* = 30° and *θ* ranging from 15° to 40°.

At this point, it would be useful to compare the performance of these lightweight hierarchical 3D rotating unit metamaterials with other 3D auxetic metamaterials found in the literature. In **Figure** **8**, low‐volume fraction BCC, FCC and TC hierarchical systems are compared with 3D auxetic metamaterials found in the literature. These include the traditional 3D re‐entrant honeycomb, studied by Chen et al.,^[^ ^56^ ^]^ a lattice‐based 3D anti‐tetrachiral architecture suggested by Iantaffi et al.^[^ ^57^ ^]^ and a re‐entrant‐based hierarchical class of 3D auxetic metamaterials proposed by Yang et al.^[^ ^58^ ^]^ These three classes of metamaterials where chosen on the basis of two factors. The first is the fact that they utilized an analogous approach to the one employed in this work to evaluate the mechanical properties, i.e., Poisson's ratios and Young's moduli were studied while considering the volume fraction (or relative density) of the overall metamaterial geometry. The second is that the geometries in question exhibit a similar range of Poisson's ratios to the FCC, BCC and TC hierarchical rotating unit metamaterials. The traditional 3D re‐entrant system and anti‐tetrachiral lattice exhibit a Poisson's ratio between −0.9 and −0.2 while the 3D hierarchical re‐entrant systems proposed by Yang et al.^[^ ^58^ ^]^ are highly anisotropic (similar to the systems proposed in this work) and exhibit a similar, albeit slightly reduced ranged, of Poisson's ratios varying from −1.2 to +0.5 depending on the loading direction and geometric configuration employed. It is evident from the plot in Figure 8, that upon comparison with BCC, FCC and TC hierarchical systems with similar volume fractions, *V_f_*, the effective Young's modulus, E_i_^*^, of the structures found in literature is significantly lower, demonstrating that the hierarchical 3D rotating auxetic systems presented in this work exhibit improved E^*^/*V_f_* ratios. In this case, the comparison was conducted with the systems with *r*/*l* ratios of 0.025 (results shown in Figure 5) which possess a similar *V_f_* to the results presented by Yang et al.,^[^ ^58^ ^]^ but as shown in Figure 6, the E^*^/*V_f_* ratio can be further improved for systems with thicker ligaments and hence, larger volume fractions.

Before concluding, it is important to highlight the significance of this work. Rotating unit metamaterials are traditionally considered to be high‐density systems which exhibit large stiffness and small strain‐tolerance, yet in this work, we have shown that through the introduction of hierarchy, one may achieve a reduction in density as large as 90% while still retaining the auxeticity and global deformation profile of the original non‐hierarchical systems. The introduction of truss elements also results in considerable reduction of localized strains and stress concentration factors, hence improving the strain tolerance and small‐strain fatigue behavior of these systems. Most importantly, these hierarchical arrangements have also been demonstrated to have the ability to significantly improve the stiffness/density ratios of these rotating cube metamaterials. This has the potential to open up many new exciting opportunities for the implementation of rotating metamaterial structures in a variety of applications involving lightweight structures such as biomedical and aerospace systems. In the biomedical field, implants such as trabecular bone prosthetics, typically necessitate metamaterial architectures possessing extremely large porosity coupled with considerable load‐bearing capacity and ability to undergo cyclic small‐strain deformation with fracture, which makes these hierarchical rotating systems particularly ideal for this purpose, while in aerospace applications, the stiffness/density ratio of a cellular material is typically the crowning factor which determines its suitability for use. The inherent scale independence of mechanical metamaterials also means that these micro lattice systems should function in the same manner at the macroscale, meaning that these geometries can also be implemented in larger systems. Further work on advancing the findings of this study should be focused on optimizing the geometry of the interconnection region between rotating units to improve the stiffness/density ratio and strain tolerance as well as the potential incorporation of auxetic hierarchical characteristics such as re‐entrancy or chirality to further lower the Poisson's ratio. The influence of constitutive material properties on the high strain deformation of these systems also merits further detailed studies. It is also worth highlighting that we believe that this work can also act as a blueprint for the design of other 3D hierarchical rotating unit metamaterials, since the techniques utilized in this work can also potentially be applied in the future to other systems such as rotating octahedrons and tetrahedra to obtain new metamaterials with superior mechanical properties and functionalities.

## 4 Conclusion

In this work, we have proposed three new classes of 3D hierarchical rotating unit systems which retain the original auxetic properties, including giant negative Poisson's ratio, of their non‐hierarchical counterparts whilst reducing their density by as much as 90%. These systems, inspired by cubic crystal arrangements including BCC, FCC and TC framework have been shown to have the potential to also exhibit superior stiffness/density ratios in comparison to non‐hierarchical systems, making them extremely ideal for implementation in applications requiring lightweight metamaterial architectures. A wide range of geometric configurations were analyzed using FEM simulations and six prototypes were produced at the microscale using two‐photon lithography and tested in situ. These hierarchical 3D microlattice structures were demonstrated to exhibit Poisson's ratios similar to the predictions of the Finite Element simulations which were retained over a considerable strain range. It is envisaged that this study will open up new avenues for the utilization of truss‐based rotating unit auxetics as well as inspire the design of other corresponding 3D hierarchical metamaterials based on rotating unit modes.

## 5 Experimental Section

### Finite Element Simulations

The FEM simulations were conducted using the ANSYS Multiphysics software. In order to maximize computational efficacy, the systems were simulated as 1/8th of an RVE, i.e., a single rotating cube, subjected to mirror periodic boundary conditions.^[^ ^52^ ^]^ This was possible due to the fact that the RVE possess three axes of mirror symmetry perfectly aligned with principal global Cartesian directions. Each structure was meshed using the SOLID187 element (a higher order 3‐D, 10‐node element with a tetrahedral shape which has quadratic displacement behavior with three degrees of freedom and was well suited for modelling irregular meshes). Following convergence testing, a minimum mesh size of *r*/2 was used for all thin ligament systems and *r*/4 for thick ligament systems. Each structure was subjected to uniaxial loading in the *x*‐, *y*‐ and *z*‐directions separately through the application of a small imposed compressive displacement and solved linearly. The Poisson's ratios, Young's moduli, metamaterial and unit cell volume for each system were extracted. The isotropic linear constituent material properties of the resin employed for the lithography 3D printing of the experimental prototypes were used: Young's modulus of 4 GPa and Poisson's ratio of 0.4.^[^ ^47^ ^]^ In the case of the nonlinear simulations equivalent to the 3D printed prototypes, the same loading conditions and material properties were used, but a nonlinear geometric solver was used with a suitable number of substeps over 10% compressive strain. Further details on the simulation methodology is provided in the Supporting Information.

### Fabrication of Microlattice Structures

The microlattice BCC, FCC and TC structures were manufactured as 5×5×5 RVE lattices using the two‐photon lithography method through a Photonic Professional GT+, Nanoscribe GmbH printer. The commercial negative tone IP‐S photoresin (Nanoscribe GmbH) was used as constituent material (stress‐strain plots provided in Supporting Information^[^ ^59^ ^]^). The hatching and slicing distances were set to be equal to 0.5 and 1 µm respectively. Before beginning the printing process, a drop of the IP‐S resin was deposited on the surface of the Indium Tin Oxide (ITO)‐coated soda lima glass substrate. Then, the polymerization was conducted through the use of the femtosecond laser operating at a wavelength of 780 nm as well as the 25X‐objective. For all samples, the laser power was set as 90 while the galvanometric scan speed was equal to 100 mm s^−1^. Furthermore, once the printing was finished, the sample was developed by first submerging it in the propylene glycol methyl ether acetate (PGMEA) solution for 20 min and then rinsing the sample for 3 min in isopropyl alcohol (IPA).

### Testing and Digital Image Correlation Analysis

Following fabrication and post‐processing, the prototypes were subjected to compressive loading tests while still adhesively attached to the printing platform. Given the small size of the samples, an external indenter with a circular cross‐section with a diameter of 3 mm was used for this purpose. Since the maximum cross‐sectional dimension of each system was less than 1000 µm, this ensured that the loading conditions were identical to that of a standard compressive *quasi*‐static loading test. The structures were compressed at a speed of 1 µm s^−1^ for up to 80 µm (equivalent to 10% global strain) in the *y*‐direction. During loading, a high‐resolution optical camera was used to record the images of the test. These images were then analyzed using a Point‐Tracking DIC analysis which considered the central RVE from the second upper row of cells as shown in Figure 7(ii). The displacements of the points at the edges of the cell were tracked and used to obtain the engineering strains necessary to calculate the Poisson's ratio *ν_yx_* of this unit cell.

## Conflict of Interest

The authors declare no conflict of interest.

## Supporting information



Supporting Information

Supplemental Video 1

Supplemental Video 2

## Data Availability

The data that support the findings of this study are available from the corresponding author upon reasonable request.
