# Supplementary material for: Lightweight 3D Hierarchical Metamaterial Microlattices
Source: Adv Sci (Weinh). 2025 May 2;12(20):2410293. doi: 10.1002/advs.202410293 (PMC12120722; doi:10.1002/advs.202410293)
Supplement: Supplementary file 1 — Supporting Information [file ADVS-12-2410293-s002.pdf]

ADVANCED SCIENCE
Open Access

Supporting Information

for *Adv. Sci.*, DOI 10.1002/advs.202410293

Lightweight 3D Hierarchical Metamaterial Microlattices

Luke Mizzi, Krzysztof K. Dudek, Andrea Frassineti, Andrea Spaggiari, Gwenn Ulliac and Muamer Kadic*

Supplementary Information for

Lightweight 3D Hierarchical Auxetic Microlattices

Luke Mizzi, Krzysztof K. Dudek, Andrea Frassineti, Andrea Spaggiari, Gwen Ulliac, Muamer Kadic

1. Implementation of Mirror Periodic Boundary Conditions

Since these metamaterials possess three axes of mirror-symmetry which are perfectly aligned with the global Cartesian coordinate system, mirror boundary conditions may be used on 1/8th of the RVE in order to simulate these systems under periodic boundary conditions. To this end, the loads and fixes shown in the figure below were used to simulated loading in *x*-, *y*- and *z*-directions respectively. Roller fixes, blocking only one displacement degree of freedom were placed on one edge for each direction, while a uniform compressive strain was applied through a displacement on the loading edge. The nodes on the other edges were constrained through coupling equations to remain aligned at all times with the principal Cartesian axes during loading.

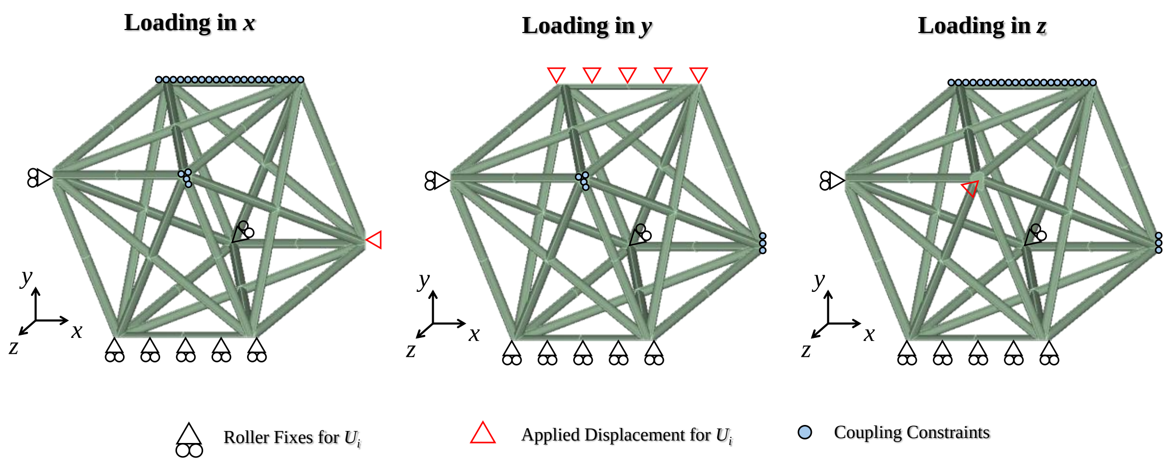

Figure S1: Mirror symmetry-based boundary conditions used to simulate systems under periodic boundary conditions.

In order to validate the accuracy of this computationally efficient simulation method, a sample number of systems were simulated as full RVEs (or unitcells) using standard periodic boundary conditions [48] and the results compared with the method described above. As shown in the

plots below, the mechanical properties obtained through both methods are identical, thus confirming the validity of the approach utilised in this work. The examples shown here are for the cases of BCC, FCC and TC systems with $r/l = 0.075$, $\theta = 30°$ and $\phi = 25°$.

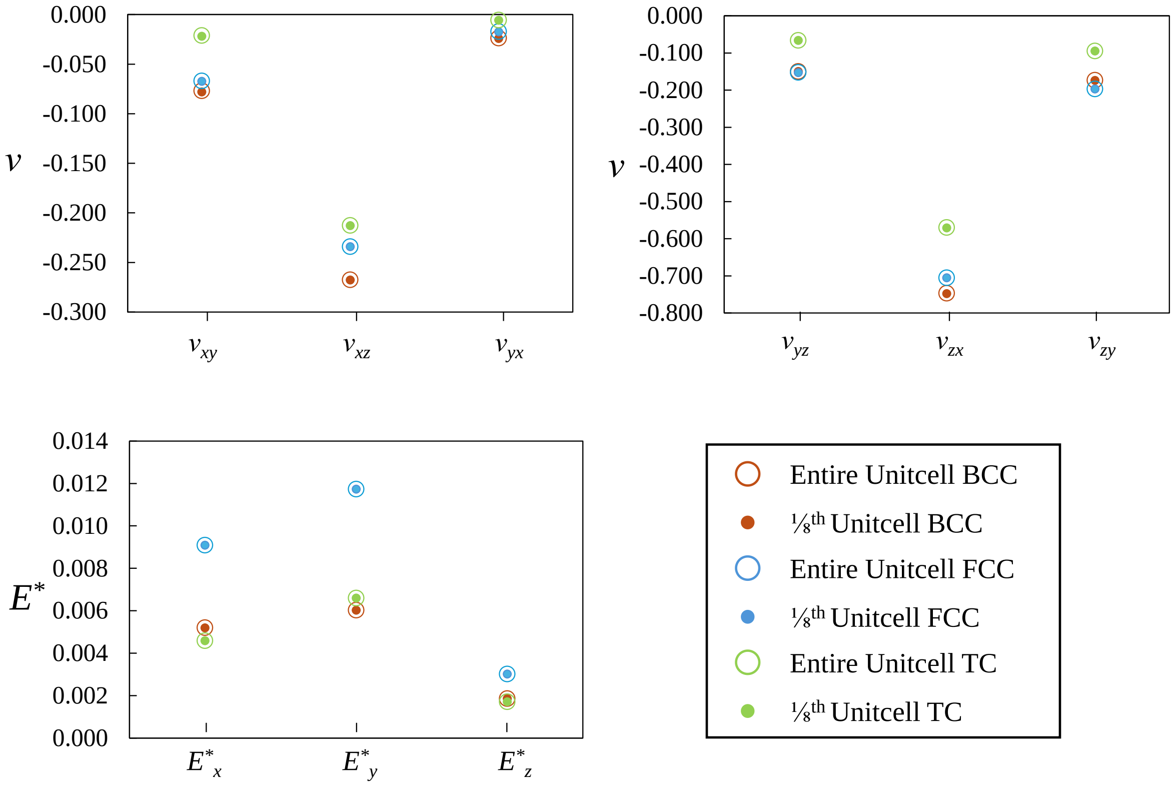

Figure S2: Comparison of the mechanical properties obtained from 1/8th unit cells with entire RVE systems. The results show identical values.

2. Influence of θ and ϕ on mechanical properties

In the plots presented in this section, the results for the variation of mechanical properties with θ and ϕ for all systems with r/l ratios = 0.025 are presented. It is clearly evident that the BCC, FCC, TC and FB systems exhibit extremely similar trends for all mechanical properties while SC demonstrate completely different behaviour. In each of the former cases, the most negative Poisson's ratios were observed for ν_{xz} with $\theta = 45°$ and $\phi = 15°$ (or 75°, both are cases are identical in this plane). On the other hand, some of the most positive values were also observed in the same plane for configurations with $\theta = 45°$ and $\phi = 45°$. This finding is not surprising, since this particular case represents the theoretically "full-opened" configuration, which is highly symmetric and rotation type deformations can only occur through instability-driven effects. For ν_{yx} and ν_{yz} the trends obtained basically mirror each other with respect to the ϕ = 45° plane. In the former case, the most negative values are observed as ϕ decreases, while for

latter, the opposite occurs, i.e. the Poisson's ratio decreases as ϕ increases. In terms of stiffness, the highest stiffness is consistently found for E^*_y when $\theta = 45°$. All of these trends are in line with theoretical predictions of the mechanical behaviour of rotating cube metamaterials.

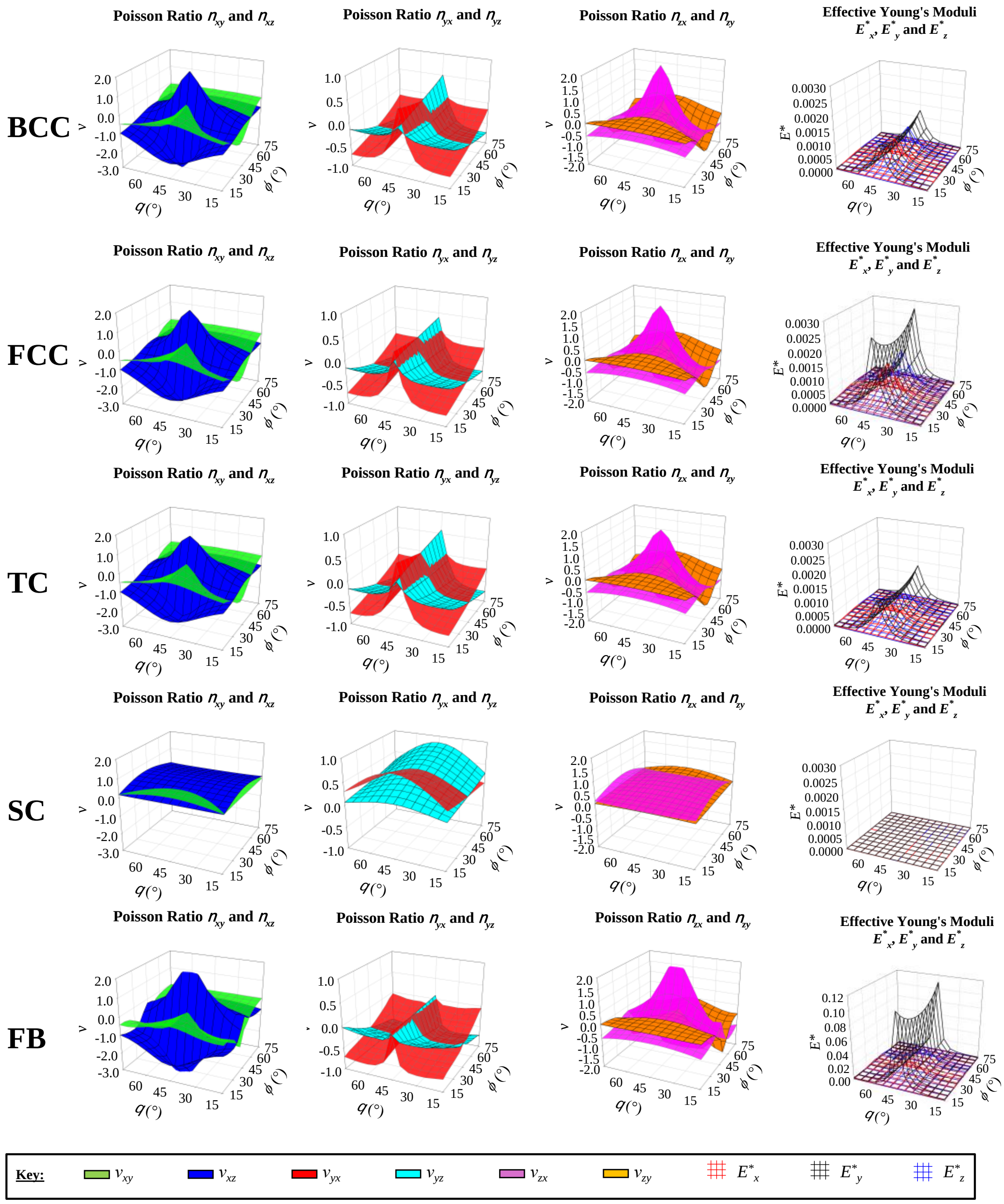

Figure S3: Mechanical properties variation for systems with $r/l = 0.025$ upon changing θ and ϕ.

3. Constituent material properties of Tough Resin

The constituent material properties of the tough resin used to construct the 3D-printed prototypes were obtained from compression tests on a 100 µm × 100 µm × 100 µm cube. The results of these tests, published previously in Chen et al. [59], yielded an elastic Young's modulus of 2.6 GPa and are replotted in the figure below:

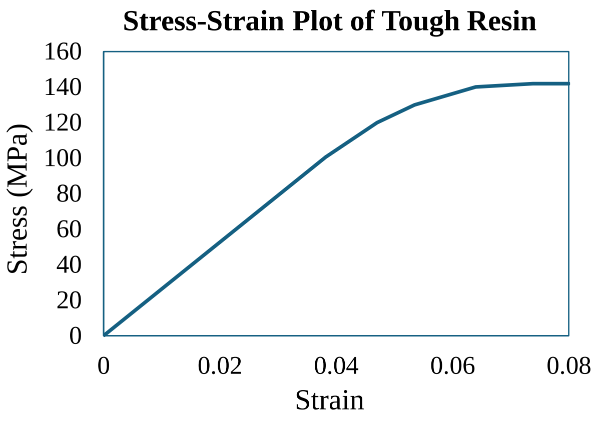

Figure S4: Stress-strain plot of Tough Resin.

4. Force-Displacement plots

The force-displacement plots obtained from the compressive loading tests on the 3D-printed structures are presented below. It is evident that, as predicted by the FEM simulations, for each set, the FCC metamaterial exhibits the highest stiffness followed by the TC and BCC systems which exhibit comparable stiffness.

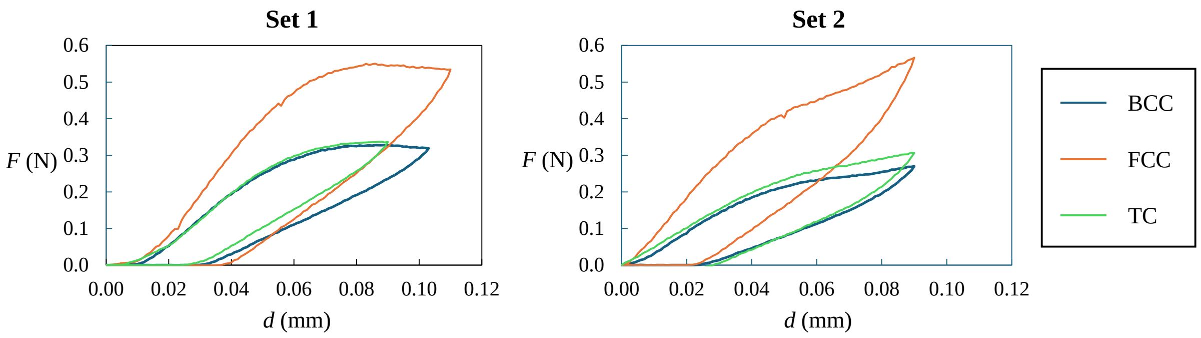

Figure S5: Force-displacement plots obtained from the experimental tests.

5. Full Plotted Results

Plots showing the entire data set of the article for Poisson's ratios, Young's moduli and Volume Fractions, divided according to ϕ values. The results shown in Figure 3 of the main article are the set listed as ϕ = 30°.

ϕ = 15°

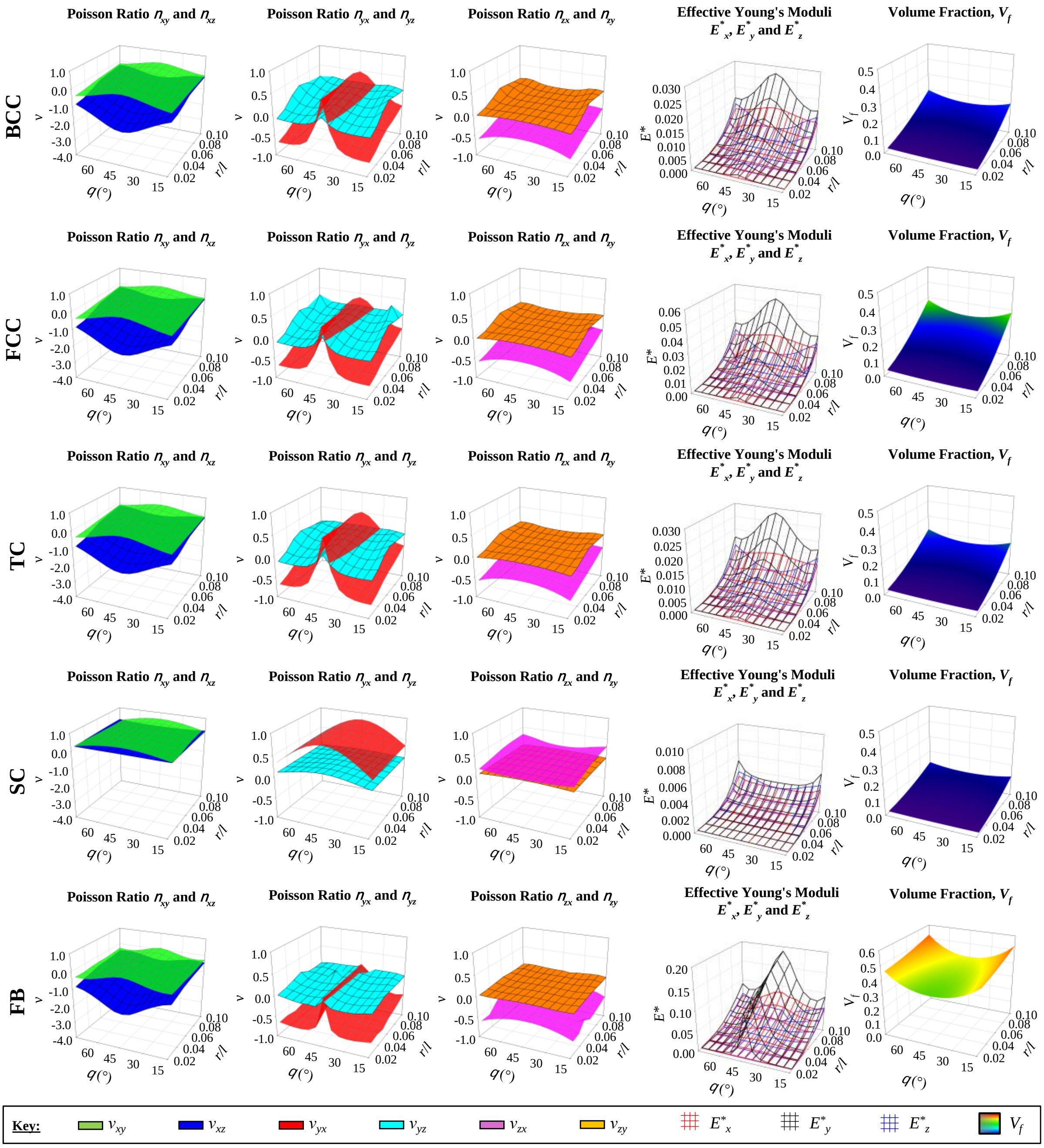

Figure S6: Mechanical properties for dataset ϕ = 15°

ϕ = 20°

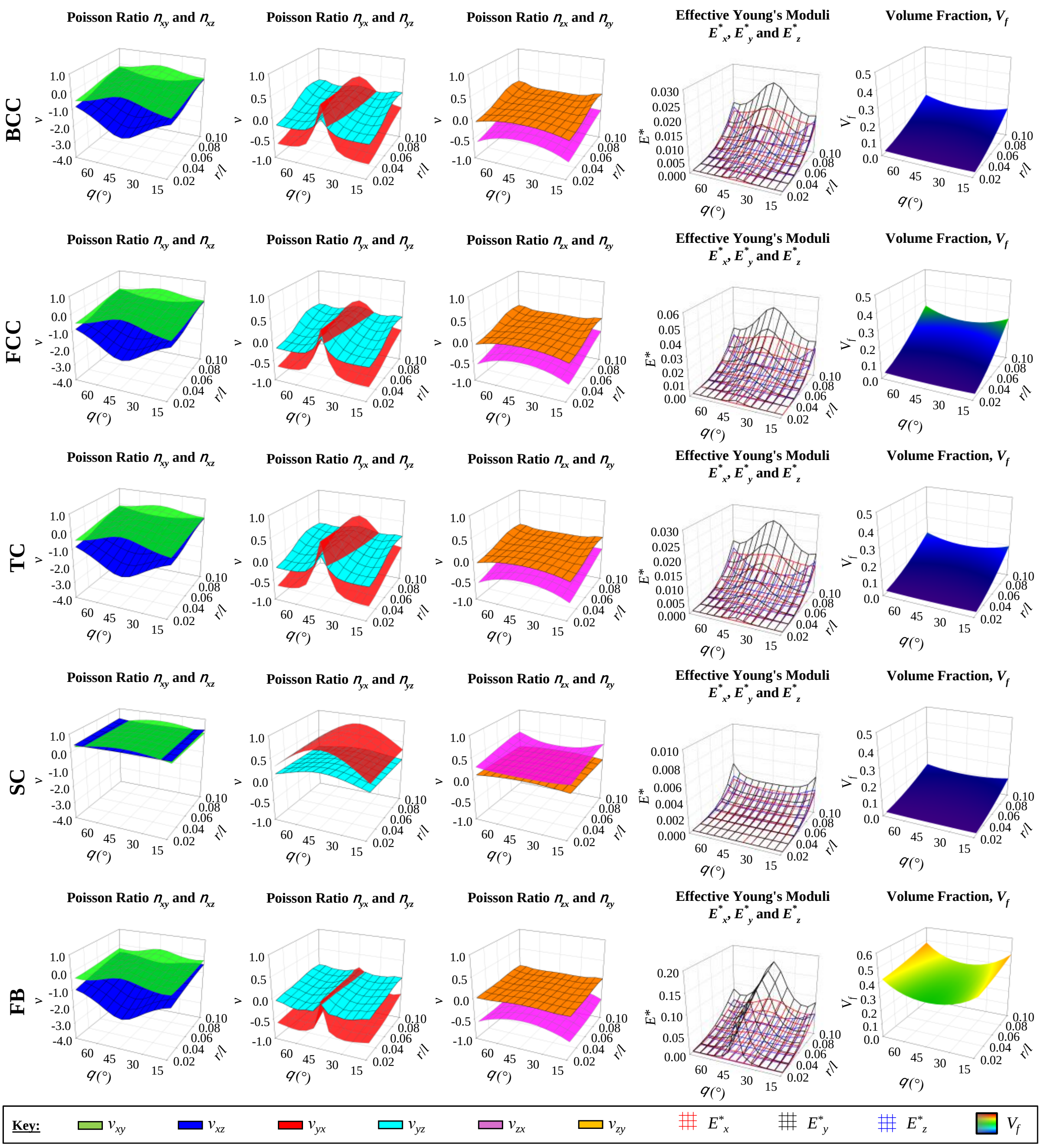

Figure S7: Mechanical properties for dataset ϕ = 20°

$\phi = 25°$

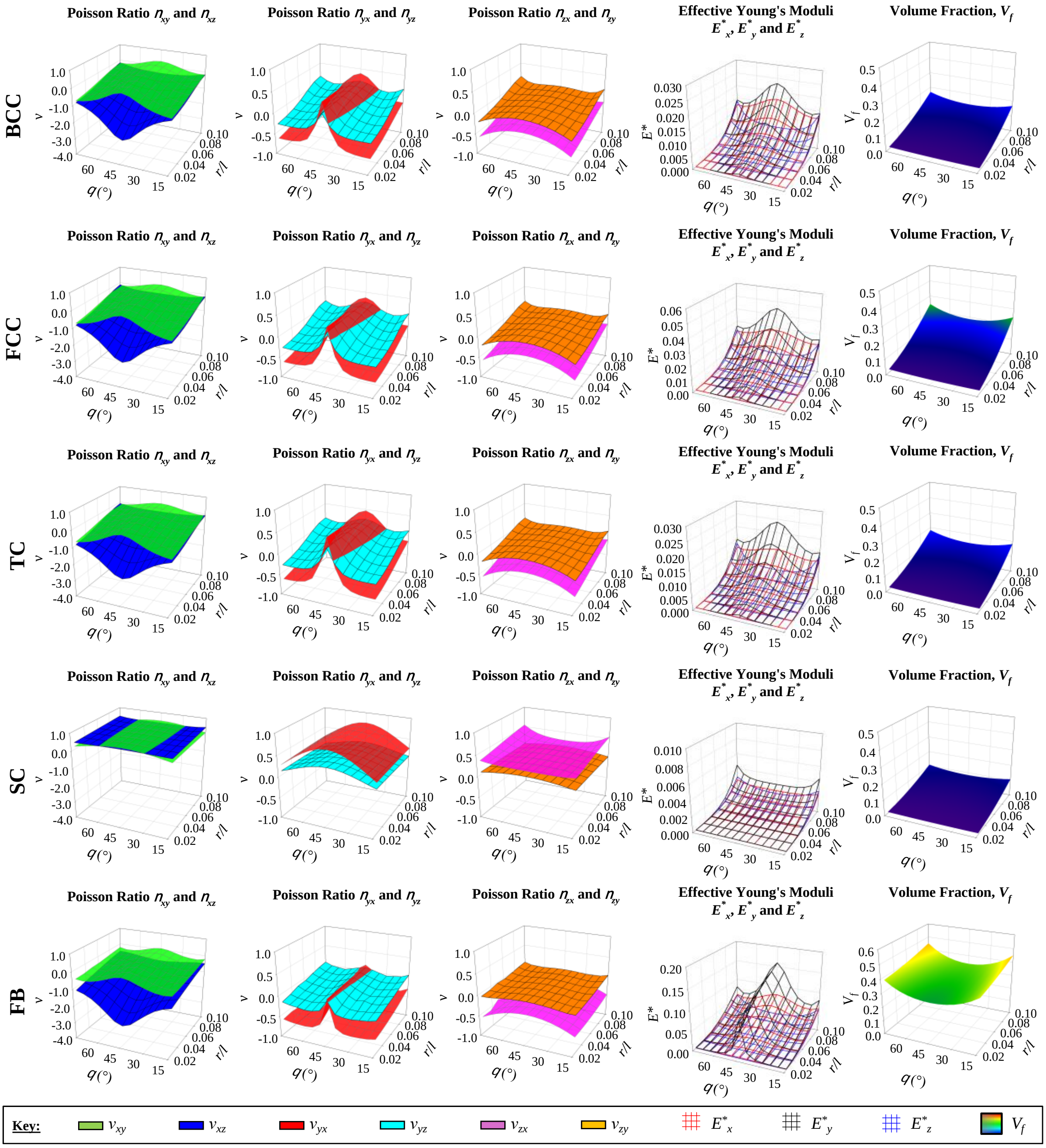

Figure S8: Mechanical properties for dataset $\phi = 25°$

ϕ = 30°

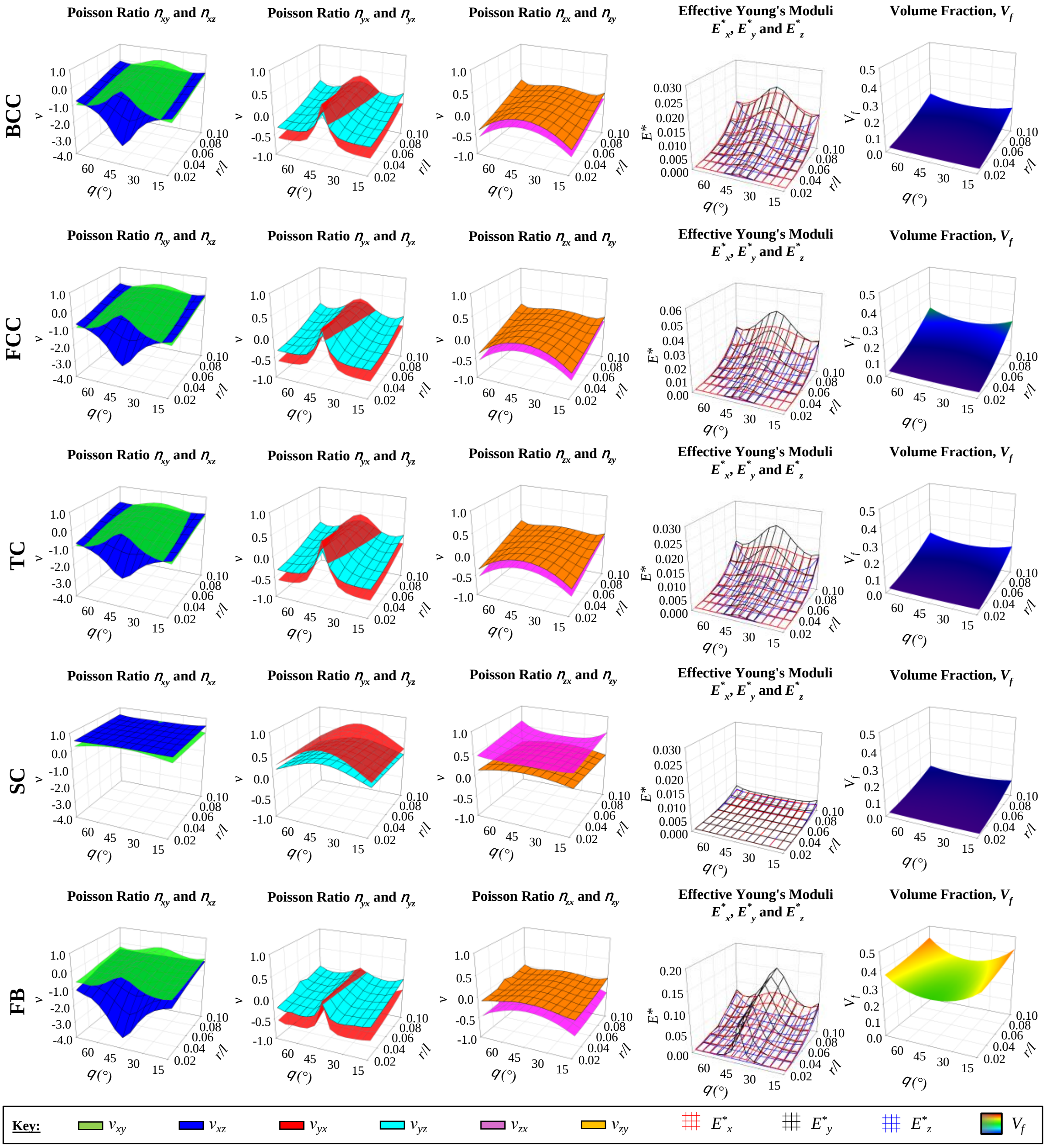

Figure S9: Mechanical properties for dataset ϕ = 30°

ϕ = 35°

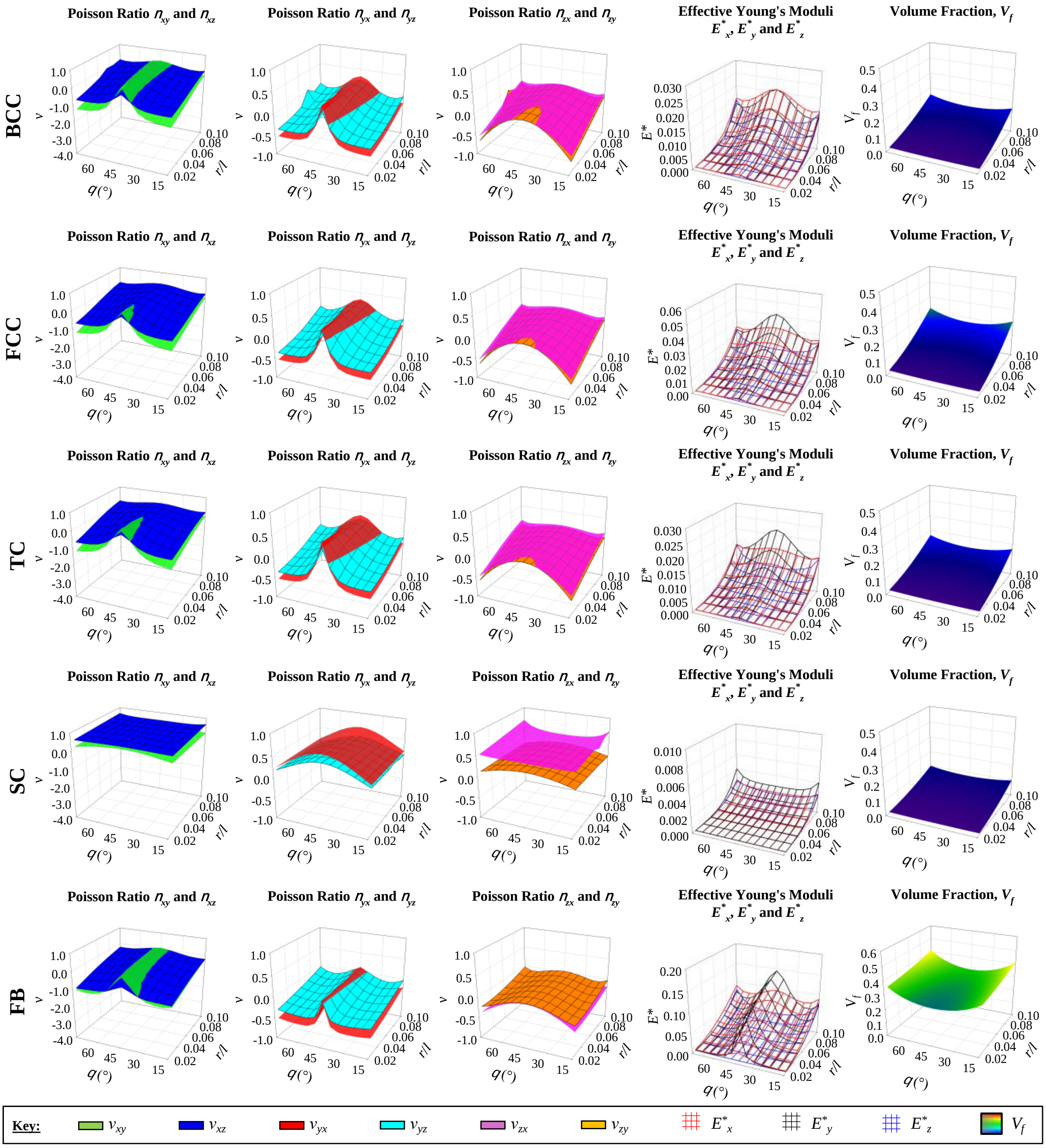

Figure S10: Mechanical properties for dataset ϕ = 35°

ϕ = 40°

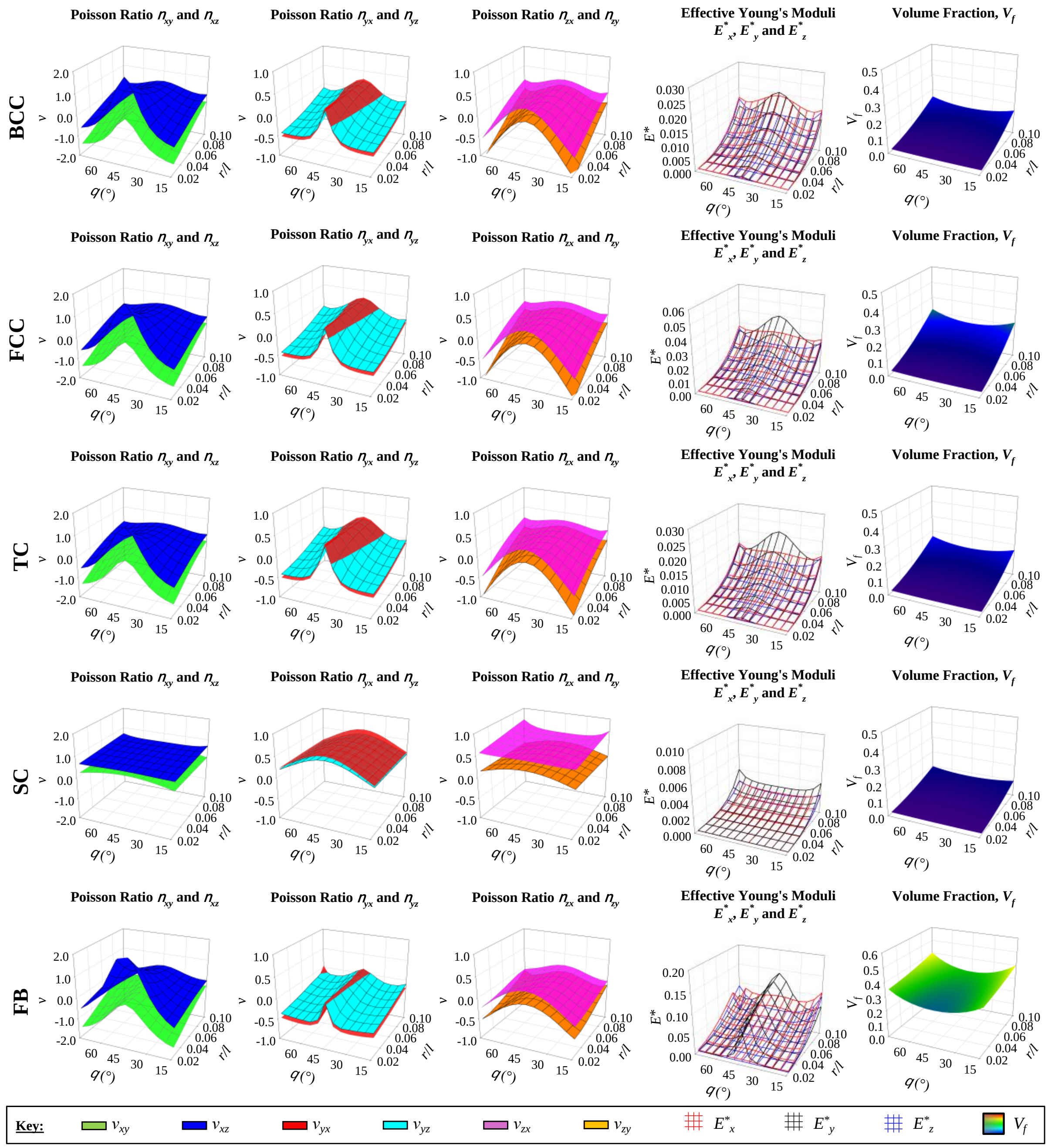

Figure S11: Mechanical properties for dataset ϕ = 40°

ϕ = 45°

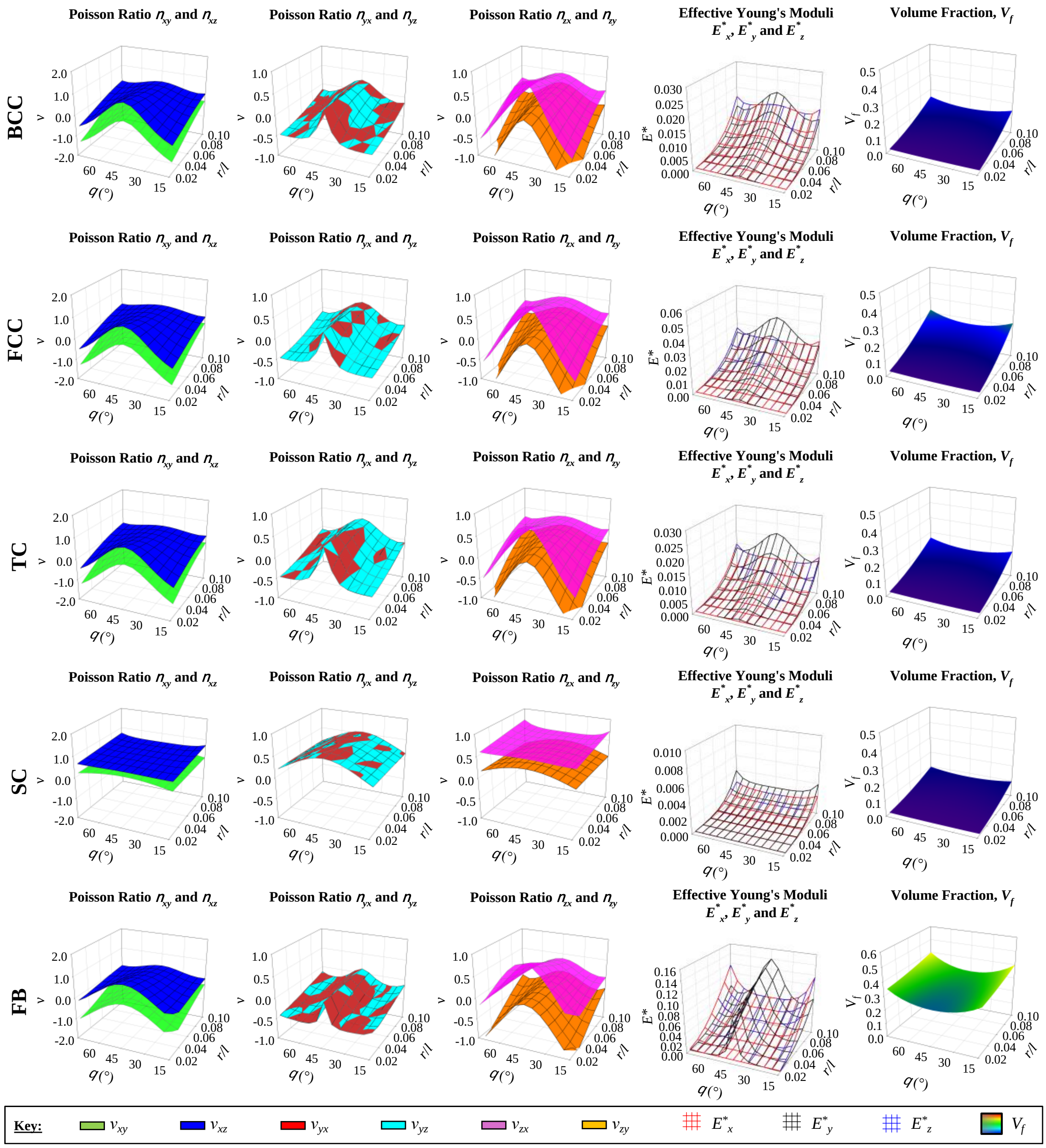

Figure S12: Mechanical properties for dataset ϕ = 45°

ϕ = 50°

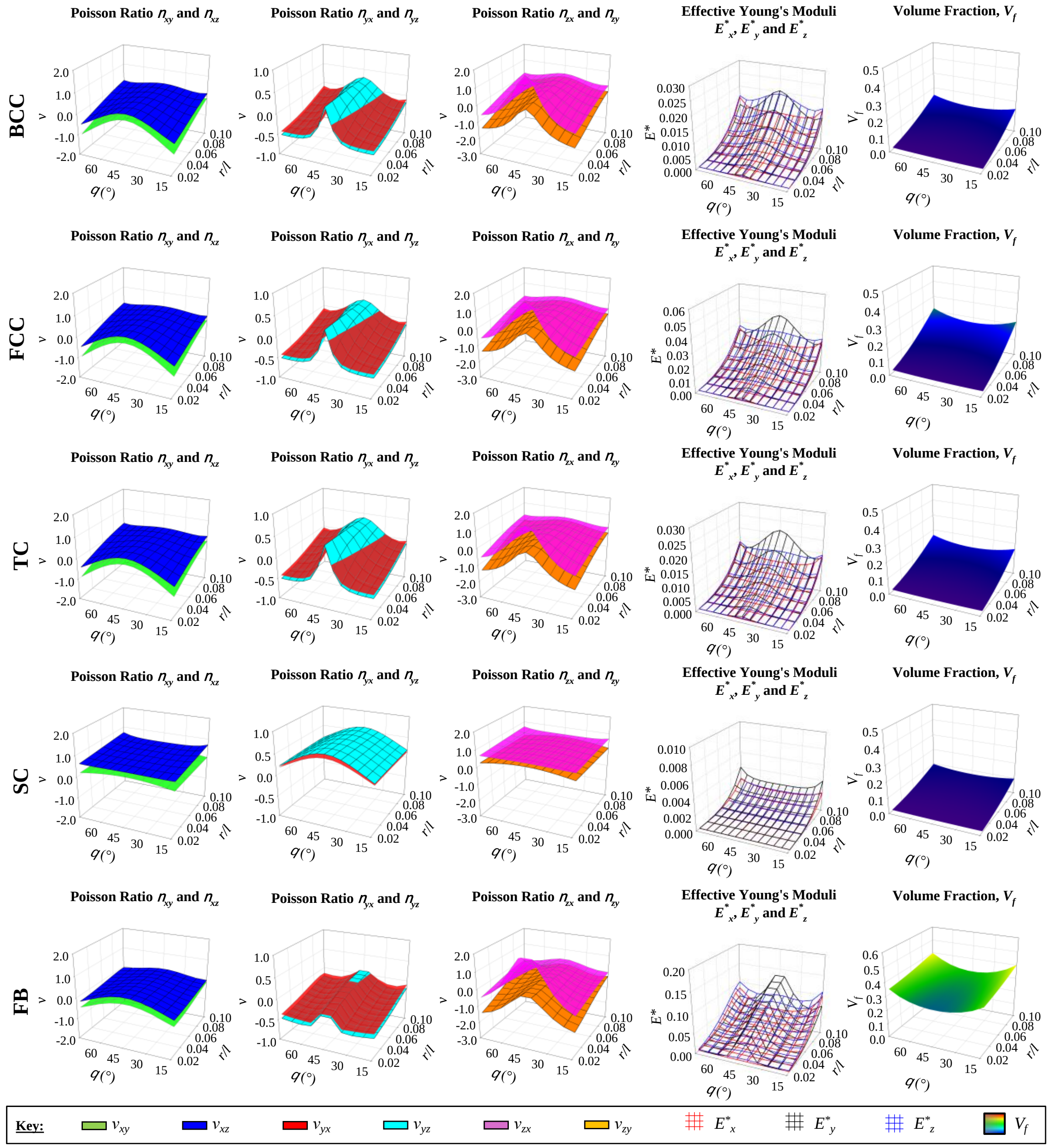

Figure S13: Mechanical properties for dataset ϕ = 50°

ϕ = 55°

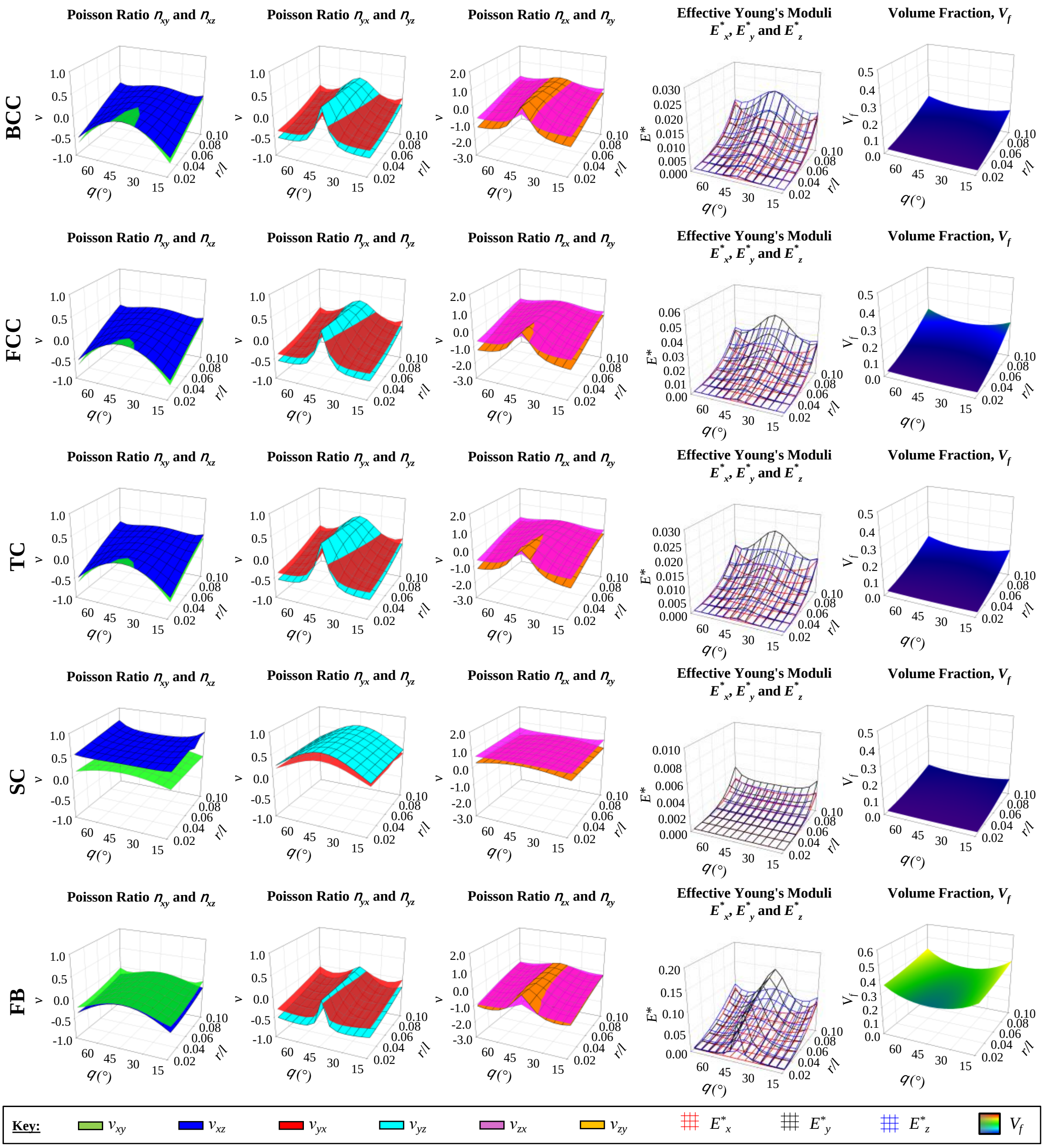

Figure S14: Mechanical properties for dataset ϕ = 55°

ϕ = 60°

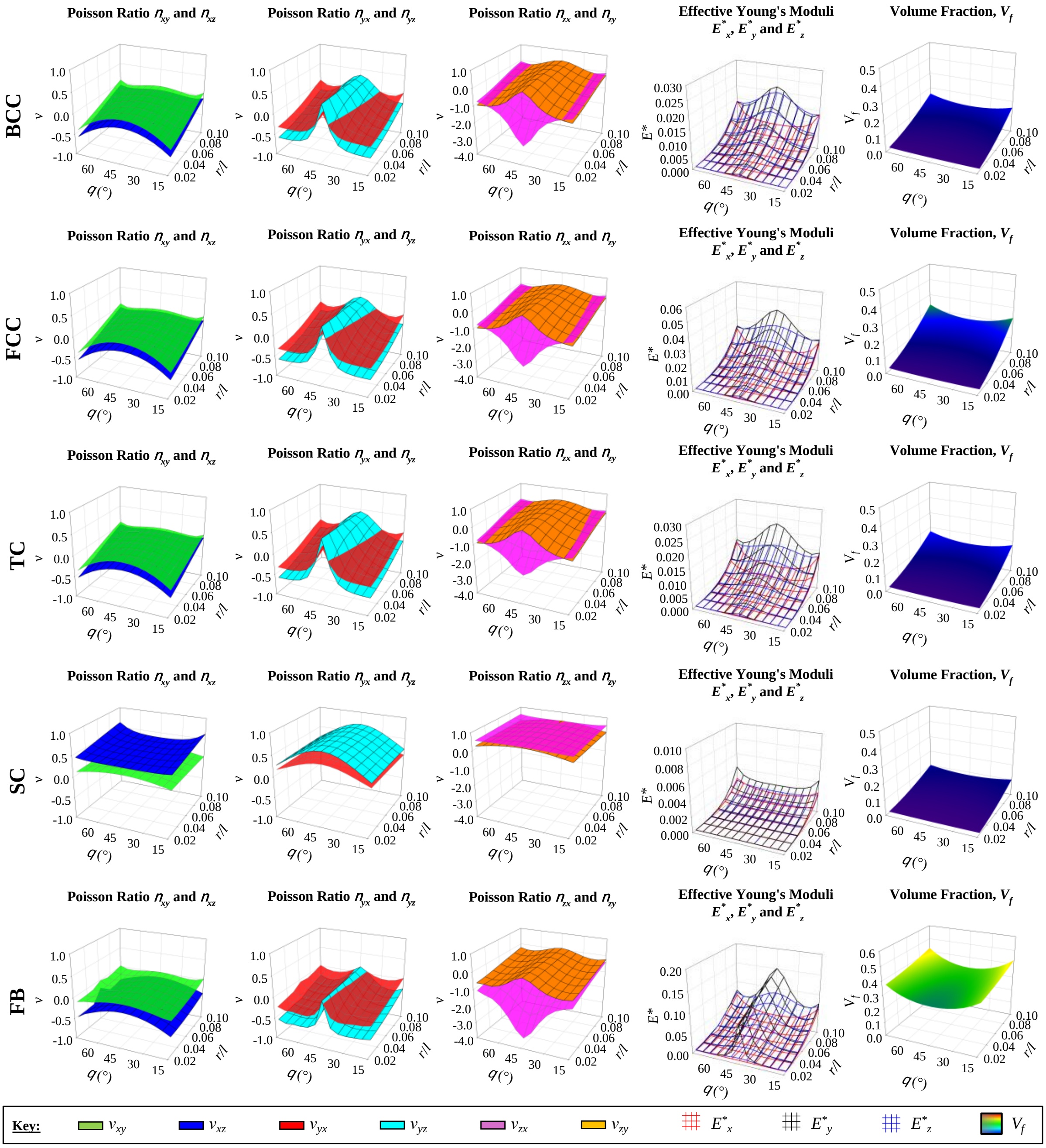

Figure S15: Mechanical properties for dataset ϕ = 60°

ϕ = 65°

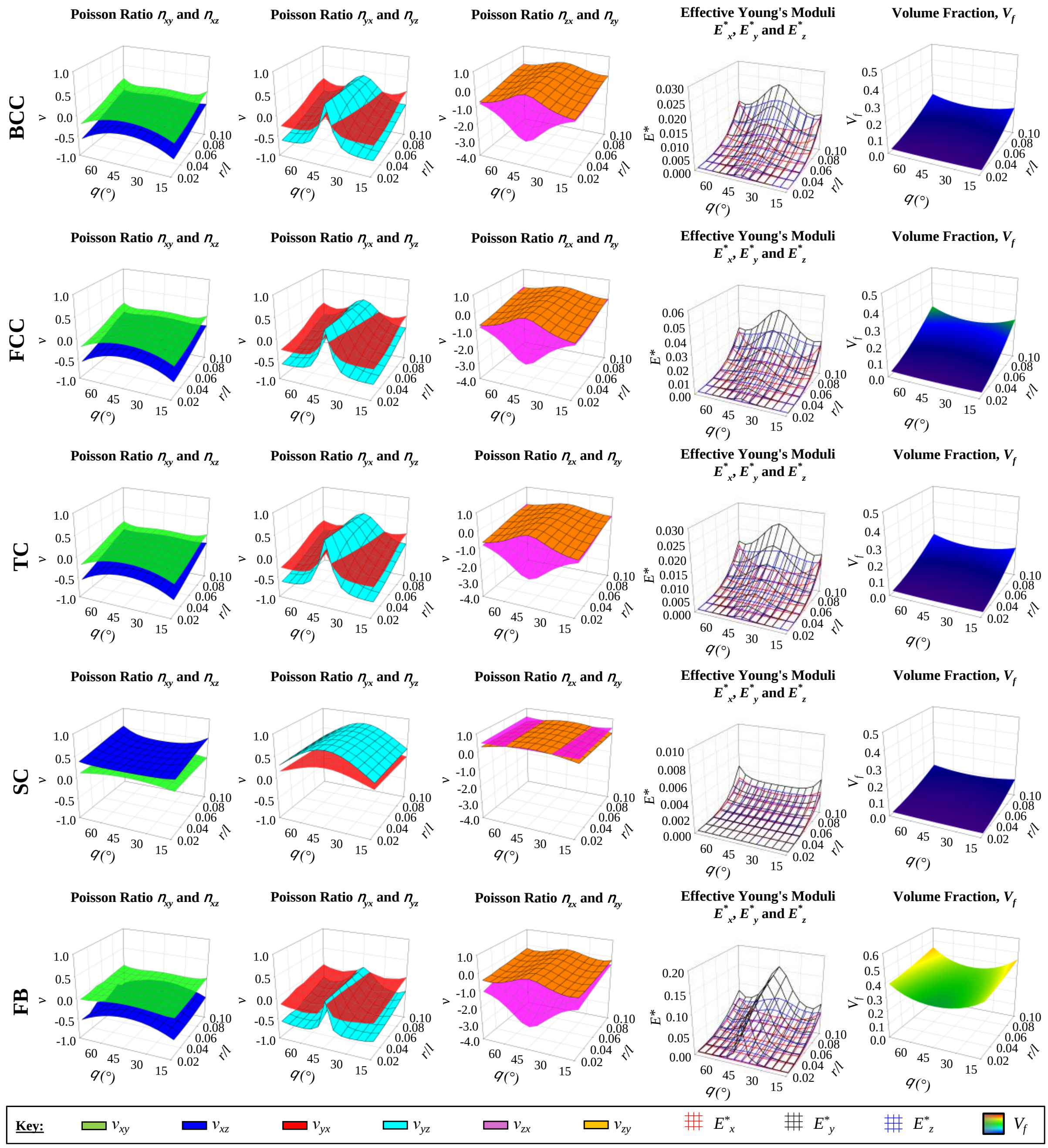

Figure S16: Mechanical properties for dataset ϕ = 65°

ϕ = 70°

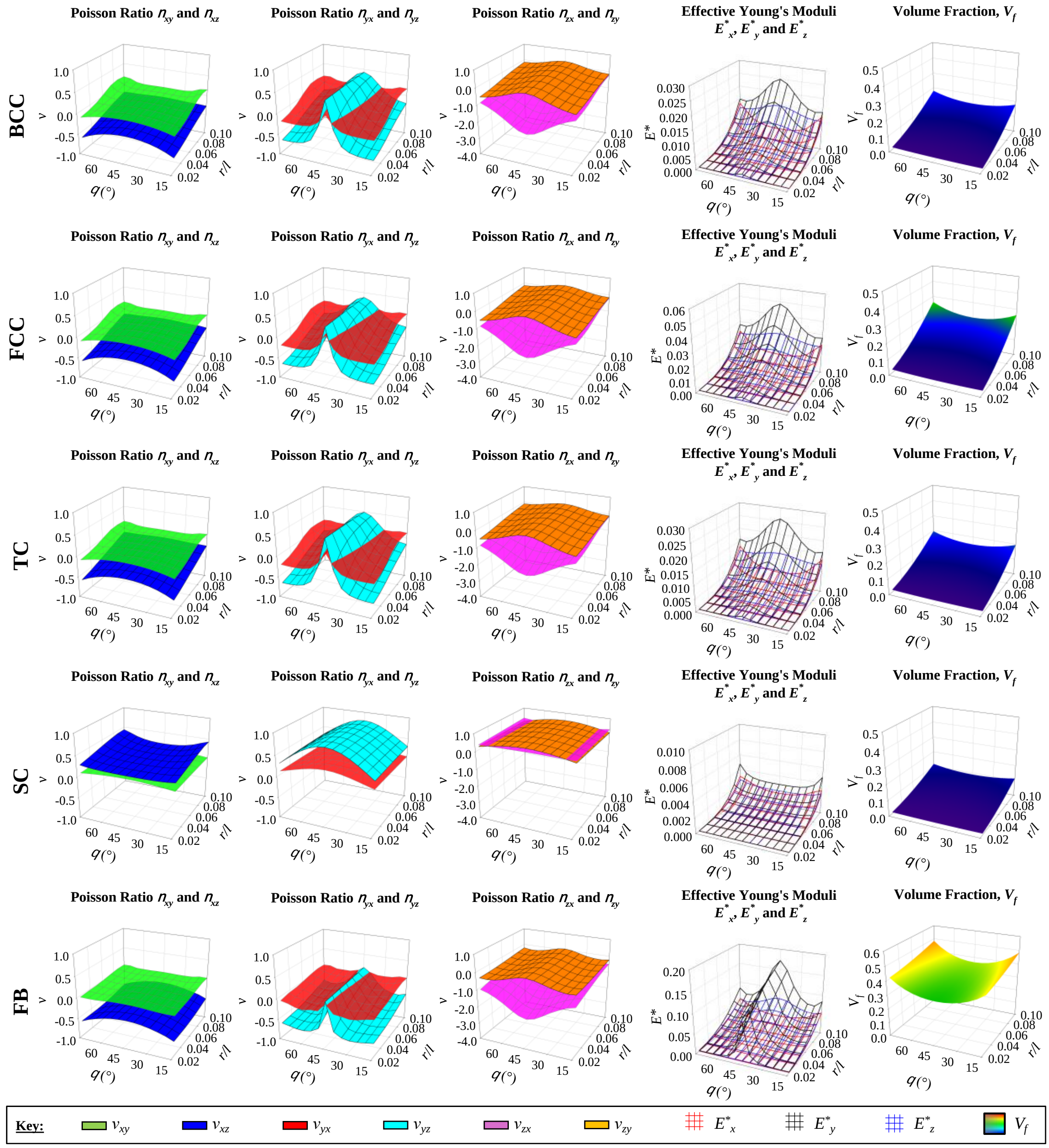

Figure S17: Mechanical properties for dataset ϕ = 70°

ϕ = 75°

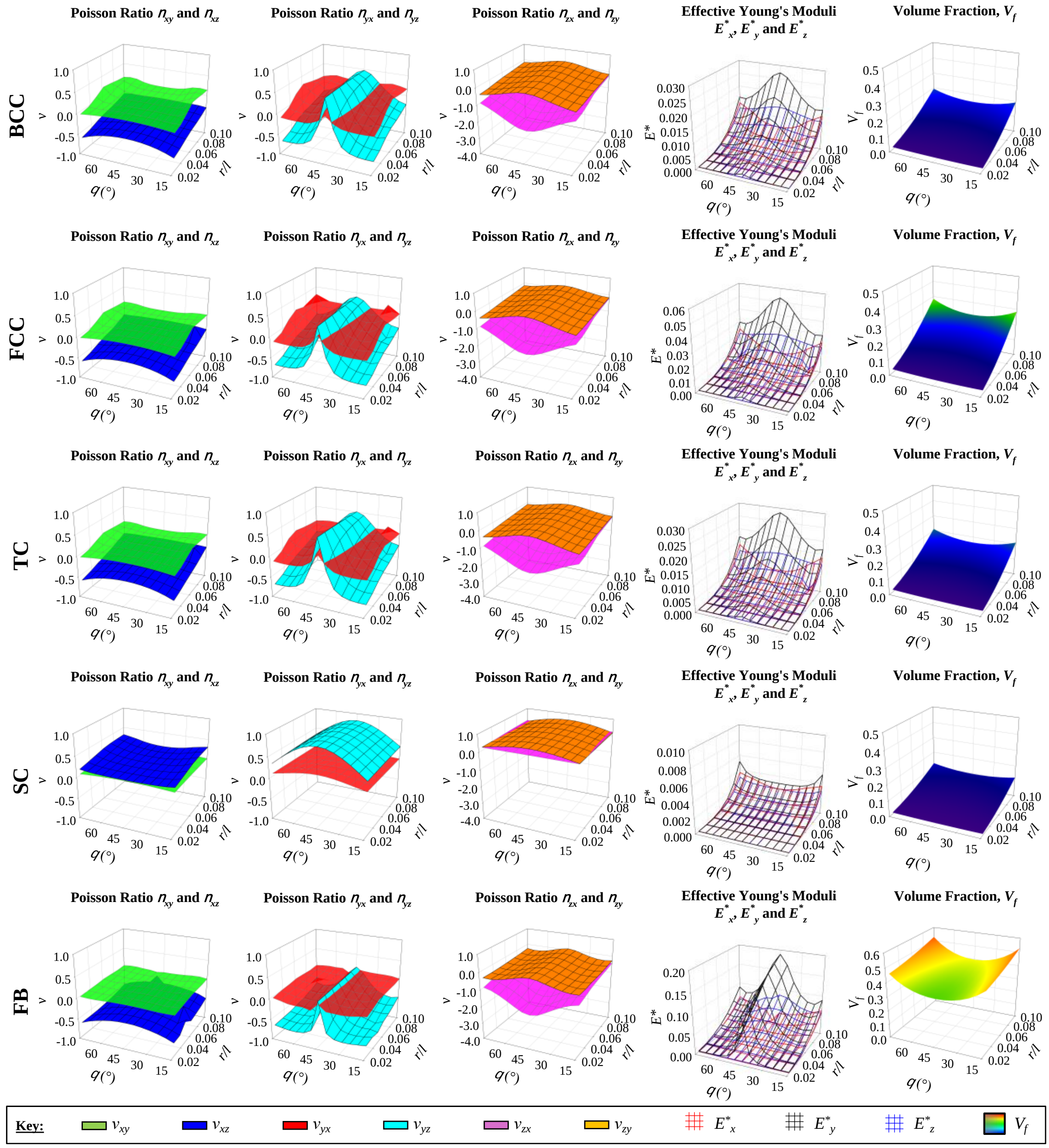

Figure S18: Mechanical properties for dataset ϕ = 75°
